# Capsular Polysaccharide Expression in Commensal *Streptococcus* Species: Genetic and Antigenic Similarities to *Streptococcus pneumoniae*

**DOI:** 10.1128/mBio.01844-16

**Published:** 2016-11-15

**Authors:** Uffe B. Skov Sørensen, Kaihu Yao, Yonghong Yang, Hervé Tettelin, Mogens Kilian

**Affiliations:** a Department of Biomedicine, Aarhus University, Aarhus, Denmark; b Beijing Pediatric Research Institute, Beijing Children’s Hospital, Capital Medical University, Xicheng District, Beijing, People’s Republic of China; c Department of Microbiology and Immunology, Institute for Genome Sciences, University of Maryland School of Medicine, Baltimore, Maryland, USA

## Abstract

Expression of a capsular polysaccharide is considered a hallmark of most invasive species of bacteria, including *Streptococcus pneumoniae*, in which the capsule is among the principal virulence factors and is the basis for successful vaccines. Consequently, it was previously assumed that capsule production distinguishes *S. pneumoniae* from closely related commensals of the mitis group streptococci. Based on antigenic and genetic analyses of 187 mitis group streptococci, including 90 recognized serotypes of *S. pneumoniae*, we demonstrated capsule production by the Wzy/Wzx pathway in 74% of 66 *S. mitis* strains and in virtually all tested strains of *S. oralis* (subspecies *oralis*, *dentisani*, and *tigurinus*) and *S. infantis*. Additional analyses of genomes of *S. cristatus*, *S. parasanguinis*, *S. australis*, *S. sanguinis*, *S. gordonii*, *S. anginosus*, *S. intermedius*, and *S. constellatus* revealed complete capsular biosynthesis (*cps*) loci in all strains tested. Truncated *cps* loci were detected in three strains of *S. pseudopneumoniae*, in 26% of *S. mitis* strains, and in a single *S. oralis* strain. The level of sequence identities of *cps* locus genes confirmed that the structural polymorphism of capsular polysaccharides in *S. pneumoniae* evolved by import of *cps* fragments from commensal *Streptococcus* species, resulting in a mosaic of genes of different origins. The demonstrated antigenic identity of at least eight of the numerous capsular polysaccharide structures expressed by commensal streptococci with recognized serotypes of *S. pneumoniae* raises concerns about potential misidentifications in addition to important questions concerning the consequences for vaccination and host-parasite relationships both for the commensals and for the pathogen.

## IMPORTANCE

Expression of a capsular polysaccharide is among the principal virulence factors of *Streptococcus pneumoniae* and is the basis for successful vaccines against infections caused by this important pathogen. Contrasting with previous assumptions, this study showed that expression of capsular polysaccharides by the same genetic mechanisms is a general property of closely related species of streptococci that form a significant part of our commensal microbiota. The demonstrated antigenic identity of many capsular polysaccharides expressed by commensal streptococci and *S. pneumoniae* raises important questions concerning the consequences for vaccination and host-parasite relationships both for the commensals and the pathogen.

## INTRODUCTION

Among the mitis group streptococci, *Streptococcus pneumoniae* (the pneumococcus) is a major human pathogen, while other species of this group are upper respiratory tract commensals that only occasionally cause infections when passively introduced into the bloodstream of humans with predisposing conditions (1). The presence of a capsule is a *sine qua non* of pneumococcus virulence. Except for conjunctivitis, noncapsular (“rough”) strains rarely cause infections. Although the mechanisms are incompletely understood, the capsule reduces complement deposition and conceals subcapsular antigens, thereby preventing clearance by phagocytosis and by mucus interactions (2, 3). Survival of the population of pneumococci in their constant competition with the human adaptive immune system is enhanced by the extensive structural diversity of the capsular polysaccharide resulting in the currently known 97 capsular serotypes (4). The individual serotypes differ with regard to experimental virulence in mice and disease outcome and prevalence in humans, which do not necessarily follow their carriage prevalence (5–9). The current conjugated vaccines against pneumococcus infections include from 10 to 13 of the most prevalent types (4).

The genetic basis of biosynthesis of 90 of the capsular polysaccharides and their structural diversity in *S. pneumoniae* was mapped by Bentley and coworkers (10). With the exception of the serotypes 3 and 37, all pneumococcus capsules are synthesized by enzymes encoded by a genetic locus (*cps*) located between the genes *dexB* and *aliA* in the genome, with occasional contribution of transferases whose genes are elsewhere in the genome. The locus consists of 12 to 20 genes encoding four conserved regulatory proteins, an initial sugar transferase, several glycosyltransferases, a polymerase (Wzy), and a flippase (Wzx), and in some cases phosphotransferases, acetyltransferases, and pyruvyltransferases. As a signature of horizontal transfer of *cps* genes between strains, all pneumococcal *cps* loci harbor several transposase genes (10). Remarkably, a total of 1,973 genes with predicted function were identified in the *cps* loci of the first 90 serotypes (10, 11). A similar Wzy/Wzx-dependent pathway is widely used in pathogenic bacteria for synthesis of cell wall polysaccharides, including lipopolysaccharides, capsular polysaccharides, extracellular polysaccharides, and glycosylation of certain surface glycoproteins (12).

It has been generally assumed that commensal bacteria that colonize mucosal membranes do not express capsular polysaccharides. However, other mitis group streptococci, including *Streptococcus oralis*, *Streptococcus sanguinis*, *Streptococcus gordonii*, and *Streptococcus mitis*, may produce several extracellular polysaccharides, including simple glucans that serve as nutritional storage and matrix in biofilms and more structurally complex polysaccharides (13–17). Some of the latter were shown to function as ligands in pilus-mediated interspecies interactions during oral biofilm formation and have been referred to as coaggregation receptor polysaccharides (CRPs) (17, 18). At least some of the CRPs are synthesized by an operon of genes similar to that of pneumococcal *cps* loci (19–23). An operon involved in the biosynthesis of capsular or coaggregation receptor polysaccharides was demonstrated in the type strain of *S. mitis* (24) but is absent in the first reported complete *S. mitis* genome (25). In our subsequent study, 12 out of 15 *S. mitis* genomes included a complete *cps* locus, one of which was identical to that of *S. pneumoniae* serotype 19C (26). Systematic studies of other mitis group species have not been performed (10, 18, 27).

The extensive structural diversity of pneumococcus capsular polysaccharides and their genetic basis have been an enigma in view of the otherwise genetically conserved pneumococcal genome. Capsular switching by genetic transformation is a common phenomenon in the population of pneumococci (28–30), and mutations may also lead to a change of serotype (31, 32). However, such genetic events cannot explain the complexity of the gene structure of *cps* loci. In a recent study, we demonstrated evidence in support of the conclusion that this diversity evolved by pneumococcal import of genes relevant to polysaccharide biosynthesis from a range of commensal streptococci (26).

Different polysaccharides have been described for selected strains of *S. oralis* and *S. mitis* (16, 17), but their structural variation within the species has not been examined systematically (33). Interestingly, serological cross-reactions between pneumococci and other streptococci of uncertain identity were observed in the past (34–37). To improve the knowledge of cell surface polysaccharides and their genetic basis, we performed comparative immunochemical and genetic analyses of capsular polysaccharides of selected species of streptococci identified according to current taxonomic principles. The aim was to examine and compare the expression of capsular polysaccharides in *S. pneumoniae* and the commensal species *S. mitis*, *S. oralis* (including the subspecies *tigurinus* and *dentisani*), and *S. infantis* as well as other mitis and anginosus group streptococci to understand their functional significance and the potential impact of immunogenic antigens shared by pathogenic and commensal bacteria.

## RESULTS

### Bacterial suspensions.

After stabilization with formaldehyde, the cells of strains of commensal species used for immunization formed aggregates, in contrast to suspensions of encapsulated pneumococci. The subsequent treatment of the bacterial suspensions with proteinase K dissolved these aggregates, indicating that proteins protruding through the capsular polysaccharide caused the autoaggregation.

### Characterization of nonpneumococcal mitis group antisera.

**FIG 1:**
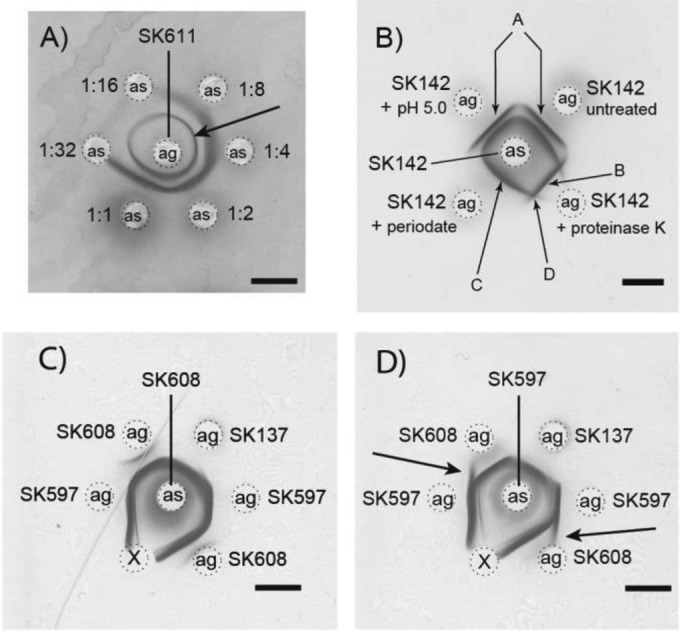
Determination of titers and specificities of mitis group antisera examined by double immunodiffusion. (A) Crude mutanolysin-lysozyme extract (antigen [ag]) of *S. mitis* strain SK611was added to the central well. Homologous antiserum (as) of SK611 (second bleeding, diluted as indicated) was added to the surrounding wells. The inner sharp line represents an unidentified protein antigen (arrow), while the outer diffuse line represents the capsular polysaccharide. The highest serum dilution that precipitates the polysaccharide was 1:8 (i.e., the titer of this antiserum is 8). (B) Precipitation lines were identified as follows. SK142 antiserum was added to the center well, and lysates made from homologous cells of *S. mitis* strain SK142 (antigen) were added to the surrounding wells as indicated. The crude lysate (untreated, mutanolysin-lysozyme extract; upper wells) contained two different antigens (arrows A) precipitated by the antiserum. The two antigens were distinct and did not cross-react (arrow D). Acetate buffer (pH 5.0) had no influence on the reactions (control well, upper left). Proteinase K treatment (lower right) digested the band closest to the center well, while the outer band (arrow B) was resistant to the proteinase. In contrast, sodium metaperiodate (lower left) decomposed the outer band, while the inner band was resistant to this treatment (arrow C). Thus, a polysaccharide antigen formed the outer band, while an unidentified protein antigen formed the inner band. (C and D**)** Example demonstrating cross-reaction or identity between capsular polysaccharide antigens prepared from different *S. mitis* strains. The center wells contained antiserum, and the surrounding wells contained crude mutanolysin-lysozyme extract (antigen) as indicated. Antigens from all three strains precipitated by antiserum SK608 showed identity. Antigens from SK597 and SK137 precipitated by antiserum SK597 showed identity, while this antiserum revealed nonidentity (arrows) between polysaccharide antigens prepared from the two strains SK597 and SK608. Wells marked with “X” contained buffer only (negative control). Bars, 5 mm.

All rabbits immunized with 12 strains of *S. mitis* and *S. oralis* selected for having a *cps* locus responded well with high levels of antibodies (titers of 32 or higher in the 3rd bleedings) that induced precipitation in immunodiffusion assays of only one or two antigens in the crude (i.e., untreated) extracts prepared from the homologous strains (exemplified in Fig. 1). Treatment of the crude antigen extracts with either sodium metaperiodate or proteinase K before testing (example shown in Fig. 1B) demonstrated that the outer line was formed by a polysaccharide antigen (protease resistant and sensitive to periodate treatment), whereas the inner line was formed by an unidentified protein antigen (protease sensitive and resistant to periodate). Based on this observation, all bacterial extracts were treated with proteinase K before use as antigens for serotyping by immunodiffusion. In this way, specificity for polysaccharide antigens was ensured and only one precipitation line was formed in each positive reaction (see examples in Fig. 1). None of the antisera reacted with group O antigen, i.e., the common cell wall polysaccharide antigen shared by *S. mitis* and *S. pneumoniae* (16), which is also known as C-polysaccharide. Therefore, the sera were considered specific for capsular polysaccharides when protease-treated extracts were used in the tests.

### Detection of *cps* locus regulatory genes by PCR.

Initially, 66 *S. mitis* strains were subjected to PCR analysis for detection of the initial regulatory gene *wzg* characteristic of the *S. pneumoniae* cps locus. Four strains of *S. pneumoniae* served as positive controls. Like the four *S. pneumoniae* controls, 40 of 66 *S. mitis* strains gave a strong reaction with both primer sets, and 9 strains gave a strong reaction with one primer set and a weaker or, in one case, negative reaction with the other primer set (selected data are shown in Table S1 in the supplemental material). Among the 66 *S. mitis* strains, 17 yielded no amplicon with either of the two primer sets. According to this finding combined with the more detailed genetic analyses (see below), 74% of these randomly selected *S. mitis* strains possessed a putatively functional *cps* locus. This may be an underestimate of the proportion of *S. mitis* strains that have a complete *cps* locus as genome sequencing of one of the PCR-negative strains revealed a complete *cps* locus.

### Serotyping of streptococcal strains.

**FIG 2:**
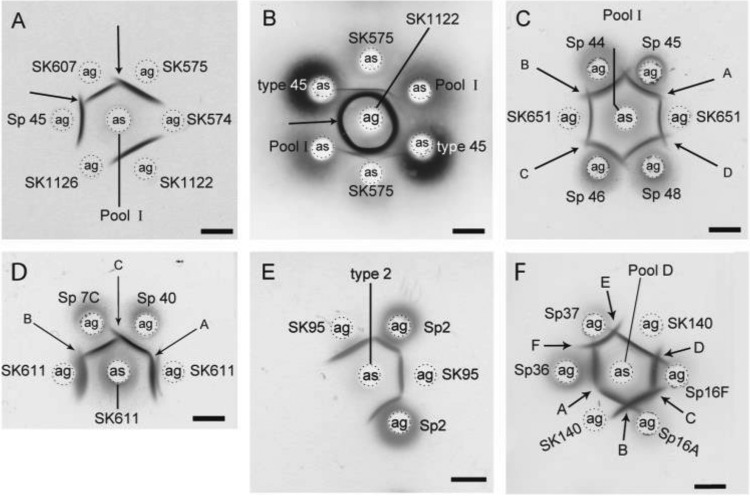
Examples of serotyping of streptococcal strains by double immunodiffusion. (A) *S. mitis* strains SK607, SK575, and SK1122 all express a polysaccharide antigen (ag) that reacts with the pneumococcal pool I antiserum (as). The SK607 polysaccharide antigen is only partially identical to polysaccharide antigens prepared from *S. pneumoniae* serotype 45 (Sp 45) and *S. mitis* SK575, respectively (arrows). (B) *S. mitis* SK1122 expresses a serotype 45 polysaccharide, as demonstrated by the identity between precipitation lines formed by the antigen and three kinds of antisera: *S. mitis* SK575, S*. pneumoniae* pool I, and type 45 antisera. (C) *S. mitis* SK651 expresses a serotype 45 polysaccharide, as demonstrated by the identity between precipitation lines formed by the SK651 antigen and pneumococcal serotype 45 capsular polysaccharide (arrow A). In contrast, SK651 differs from the pneumococcal serotypes 44, 46, and 48 (arrows B, C, and D, respectively). (D) Identity and partial identity between polysaccharide antigens prepared from *S. mitis* SK611 and *S. pneumoniae* types 7C (Sp 7C) and 40 (Sp 40). When tested against the homologous antiserum, the polysaccharide prepared from strain SK611 showed identity to pneumococcal serotype 40 polysaccharide (arrow A) and partial identity to pneumococcal serotype 7C polysaccharide (arrow B). Partial identity between the two pneumococcal serotypes 7C and 40 was also demonstrated (arrow C). (E) Identity between polysaccharide antigens prepared from *S. mitis* SK95 and *S. pneumoniae* type 2 (Sp 2) as shown by reactions with pneumococcal type 2 antiserum applied to the center well. (F) *S*. *infantis* strain SK140 was identified as type 36 by comparison with polysaccharides prepared from known pneumococcal serotypes by reaction with pneumococcal pool D antiserum. Polysaccharide prepared from SK140 shows identity to pneumococcal type 36 capsular polysaccharide antigen (arrow A) but is dissimilar from the three types 16A, 16F, and 37 (arrows B, D, and E, respectively). As expected, partial identity was observed between the two pneumococcal serotypes 16A and 16F (arrow C), while the two pneumococcal serotypes 36 and 37 were distinct from each other (arrow F). Bars, 5 mm.

Precipitations appeared in the gels when the prepared antisera were tested against polysaccharides extracted from the homologous streptococcal strain (shaded in Table S2 in the supplemental material). As reactions with the common antigen could be ruled out, this demonstrates that the 12 streptococcal strains selected for immunization all possessed a cell-wall-associated polysaccharide distinct from the common group O antigen (examples shown in Fig. 1 and 2).

Analyses were performed to explore whether the detected polysaccharides were unique or shared by unrelated streptococcal strains. Polysaccharide extracts prepared from 84 nonpneumococcal mitis group streptococci (including the strains used for immunization) and from pneumococcal strains of 90 different serotypes were examined in a checkerboard system: i.e., all extracts were examined in the 12 nonpneumococcal sera and in 14 pneumococcal diagnostic pool sera (pools A to I and P to T). Altogether, more than 4,500 tests were performed by double immunodiffusion (see representative examples in Fig. 1 and 2). Thirty-five of the 84 nonpneumococcal strains exhibited a positive reaction in one or more of the antisera (Table S2). Some of the strains showed identity to or at least cross-reaction with a known pneumococcal serotype. When possible, the serotypes were established by confirmatory double immunodiffusion tests either by comparison with antigens prepared from known pneumococcal serotypes or by the use of pneumococcal group or type sera (examples of reactions are shown in Fig. 2). Based on the results, strains were assigned to recognized *S. pneumoniae* serotypes or serogroups, to provisional new serogroups designated smI to smIV (Table S1 and Table S2), or to unique structures demonstrated in a single strain only. The characteristics of each of these serogroups are described below.

### Antigenic identity to *S. pneumoniae* serotypes. (i) Serotype 19C.

Among the *S. mitis* strains, only the homologous strain showed a positive reaction with the SK564 antiserum (Table S2). In addition, capsular polysaccharide prepared from the pneumococcal serotype 19C and 19B strains gave distinct positive reactions. Confirmatory tests revealed serological identity between the SK564 polysaccharide and pneumococcal serotype 19C (Table S2).

### (ii) Serotype 45.

Polysaccharides of nine *S. mitis* strains, SK574, SK575, SK607, SK609, SK614, SK615, SK616, SK651, and SK1122, and pneumococcal serotype 45 reacted with the anti-SK575 serum. Confirmatory tests revealed identity between capsular polysaccharides of pneumococcal serotype 45, SK575, and five of the other *S. mitis* strains (SK574, SK609, SK615, SK651, and SK1122 [Table S2]). Serological analysis of the three remaining cross-reactive strains, SK607, SK614, and SK616, revealed partial identity to serotype 45 (Table S2). Some examples of cross-reactions between *S. mitis* strains and pneumococcus serotype 45 are shown in Fig. 2C.

### (iii) Serotype 40.

Only *S. mitis* strain SK611 and pneumococcal types 40 and 7C reacted with the SK611 antiserum (Table S2). A confirmatory test revealed serological identity with the pneumococcal serotype 40 polysaccharide and confirmed the previously demonstrated partial identity to type 7C (Fig. 2D).

### (iv) Serotype 2.

*S. oralis* subsp. *dentisani* strain SK95 did not react with any of the prepared antisera but reacted in pneumococcal diagnostic pool antisera A and T. Additional tests demonstrated that the polysaccharide of *S. oralis* subsp. *dentisani* strain SK95 was serologically identical with the pneumococcal serotype 2 polysaccharide (Fig. 2E).

### (v) Serotype 36.

Immunodiffusion tests of polysaccharides extracted from *S. infantis* strains SK140 and SK1076 showed a close serological relationship to pneumococcal serotype 36 (reaction for SK140 shown in Fig. 2F). The antigenic identity was not definitively confirmed as antisera were not available for the two *S. infantis* strains (see results of the genetic analysis below).

### (vi) Serotype 21.

Two *S. mitis* strains, SK1123 and SK1124, cross-reacted with each other and with pneumococcal serotype 21 polysaccharide when analyzed with pneumococcal serum pool E (reaction not shown) (Table S2).

### Capsular polysaccharides distinct from *S. pneumoniae* serotypes. (i) *S. mitis* serogroup smI.

Immunodiffusion analyses revealed cross-reactions between the three *S. mitis* strains SK137, SK597, and SK608 (Table S2), but “spurs” at the ends of some of the precipitation lines imply minor structural differences in the three polysaccharides (Fig. 1D). Interpretation of the precipitation lines seen in Fig. 1D suggests that strains SK137 and SK597 express identical polysaccharides, while the polysaccharide of SK608 apparently lacks an epitope relative to the two other strains. Four additional *S. mitis* strains, SK135, SK138, SK602, and SK677, not used for immunization, reacted with the same three antisera (Table S2). Thus, these seven mitis strains possess similar polysaccharides, although the comparison demonstrated minor differences (Fig. 1D). Based on these results and according to the tradition for pneumococcal serology (i.e., the Kauffmann-Lund nomenclature [38]), we assigned the seven mentioned *S. mitis* strains to a “serogroup,” i.e., strains displaying extensive serological cross-reactivity due to common antigenic determinants but allowing for minor structural differences. The serogroup I strains did not cross-react with any pneumococcal capsular polysaccharide.

### (ii) *S. mitis* serogroup smII.

The SK142/NCTC 12261^T^ antiserum only reacted with the homologous strain (Table S2). No similarities to pneumococcal capsular polysaccharides were detected.

### (iii) *S. mitis* serogroup smIII.

In addition to the homologous strain, three *S. mitis* strains (SK334, SK596, and SK1073) reacted with the antiserum to the SK271 polysaccharide (Table S2). The strains of this group showed one continuous precipitation line when tested against the SK271 antiserum. None of the four strains showed antigenic similarities to pneumococcal capsular polysaccharides.

### (iv) *S. mitis* serogroup smIV.

Strain SK637 assigned to serogroup IV was unique among the strains, and no cross-reaction was observed with any pneumococcal serotype (Table S2).

### Reactions with anti-*S. oralis* SK23/ATCC 35037^T^ serum.

In addition to the homologous strain, four strains belonging to three different *Streptococcus* species (i.e., *S. mitis* [SK578 and SK646], *S. oralis* subsp. *tigurinus* [SK313], and *S. infantis* [SK959]), reacted in the antiserum raised against *S. oralis* SK23 with precipitates suggesting identity or close similarity. None of them reacted with pneumococcus typing antisera (Table S2). Since none of the strains had mutually related *cps* loci (see the results of genetic analysis), the observed reaction may have been caused by antigens unrelated to the capsular polysaccharide.

### Serologically unclassified strains.

Of a total of 84 nonpneumococcal mitis group streptococci serologically examined in this study, 49 strains reacted neither in any of the pneumococcus typing antisera nor in the 12 antisera raised against selected *S. mitis* capsular polysaccharides (Table S2). Among 49 *S. mitis* strains that showed evidence of a *cps* locus by PCR, 21 (43%) strains did not react in any of the pneumococcus typing antisera or antisera raised against selected *S. mitis* capsular polysaccharides (Table S2).

### Genetic analyses of *cps* loci.

The genomes of 22 *S. mitis*, 3 *S. pseudopneumoniae*, 10 *S. oralis* subsp. *oralis*, 5 *S. oralis* subsp. *tigurinus*, 5 *S. oralis* subsp. *dentisani* (previously “*S. mitis* biovar 2”), and 6 *S. infantis* strains, as well as the nonclassified strain ATCC 6249 (incorrectly labeled as *S. mitis*), were examined for the presence and structure of *cps* loci (Table S2). The search initially focused on the sequence between the genes *dexB* and *aliA*/*sarA*, which flank the *cps* locus in all *S. pneumoniae* serotypes and in previously examined strains of *S. mitis* (10, 26). Full *cps* loci spanning from 16,938 to 26,507 bp in length (sequence between end of *dexB* and start of *aliA*) and including the four regulatory genes *wzg*, *wzh*, *wzd*, and *wze*, glycosyltransferases, polymerase, and flippase were demonstrated in 16 of the 22 genomes of *S. mitis* strains. In the genome sequences of SK255 and SK569, the genes of the *cps* locus were present on two or three different contigs. The gap between these contigs in each of these strains was closed by Sanger sequencing of PCR amplicons of the gap regions.

**FIG 3:**
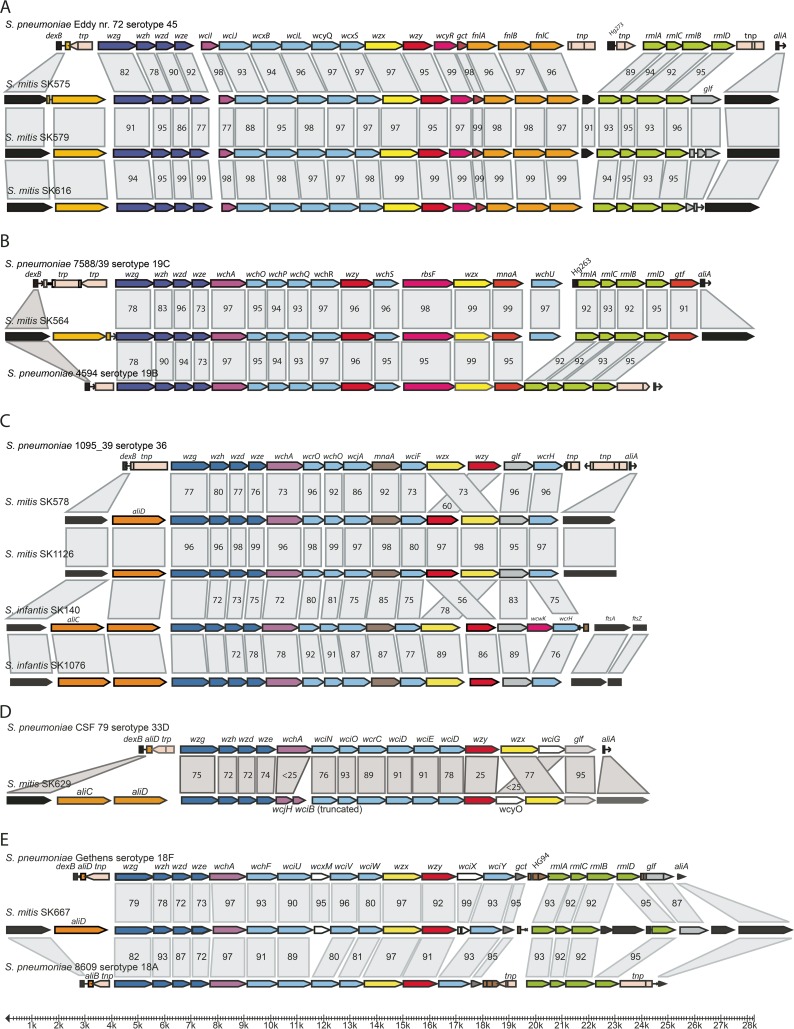

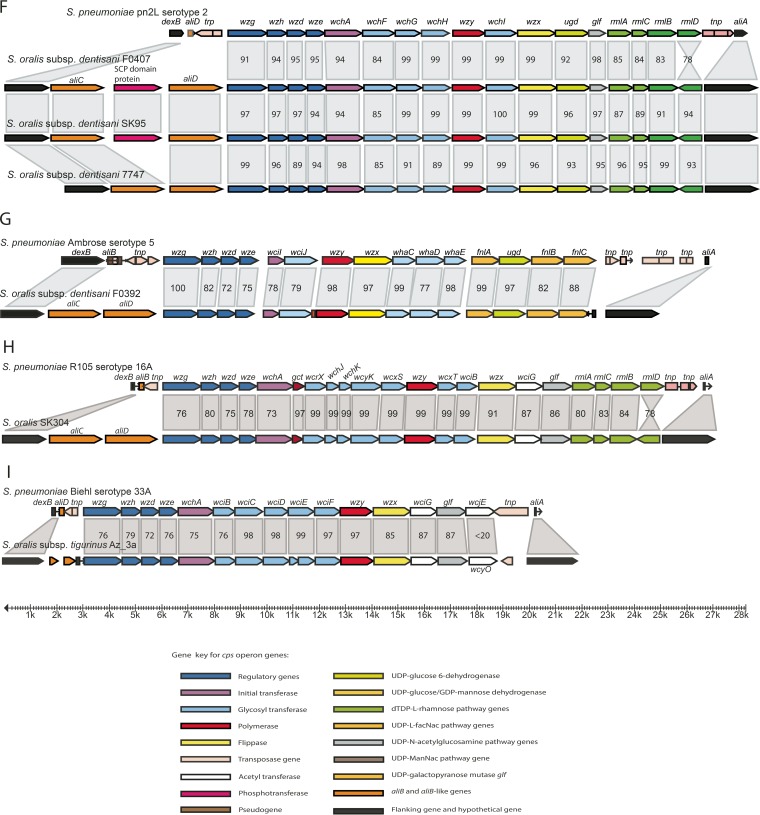
Diagrammatic representation of the capsular biosynthesis loci in commensal streptococci with complete or close identity to recognized serotypes of *S. pneumoniae*: 45 (A), 19C (B), 36 (C), 33D (D), 18F (E), 2 (F), 5 (G), 16A (H), and 33A (I). Gray boxes indicate functional identity as revealed by the annotation, and the numbers in the boxes indicate the percentage of nucleotide identity.

**FIG 4:**
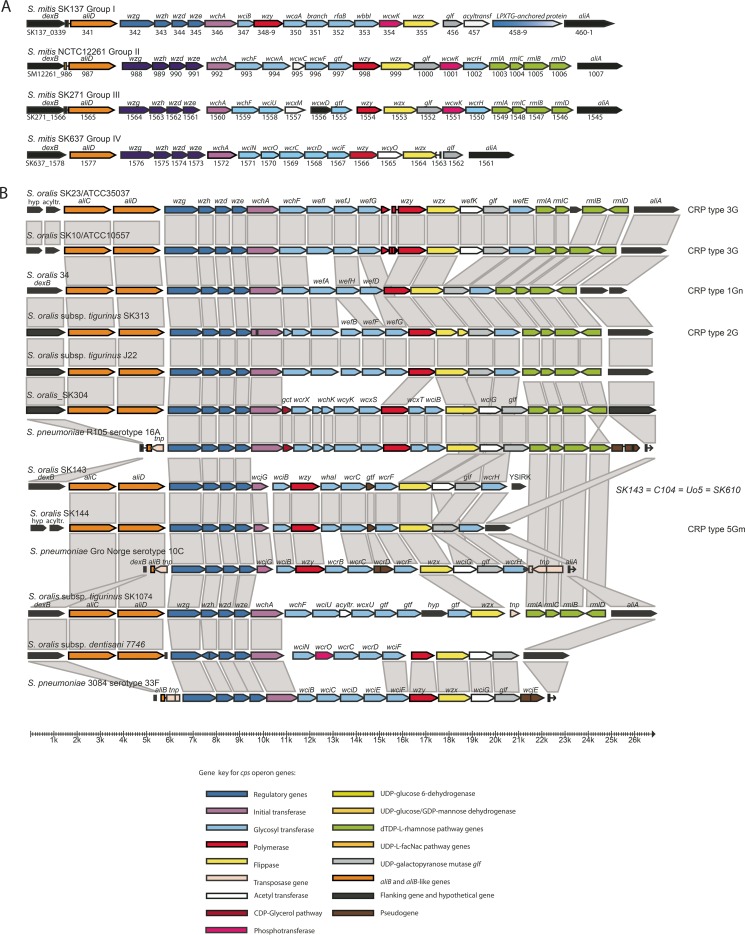
Diagrammatic representation of the capsular biosynthesis loci in commensal streptococci distinct from recognized serotypes of *S. pneumoniae*. Gray boxes indicate functional identity as revealed by the annotation. (A) Strains of *S. mitis*. (B) Strains of *S. oralis*. The corresponding coaggregation receptor polysaccharide (CRP) type designations are listed to the right.

**FIG 5:**
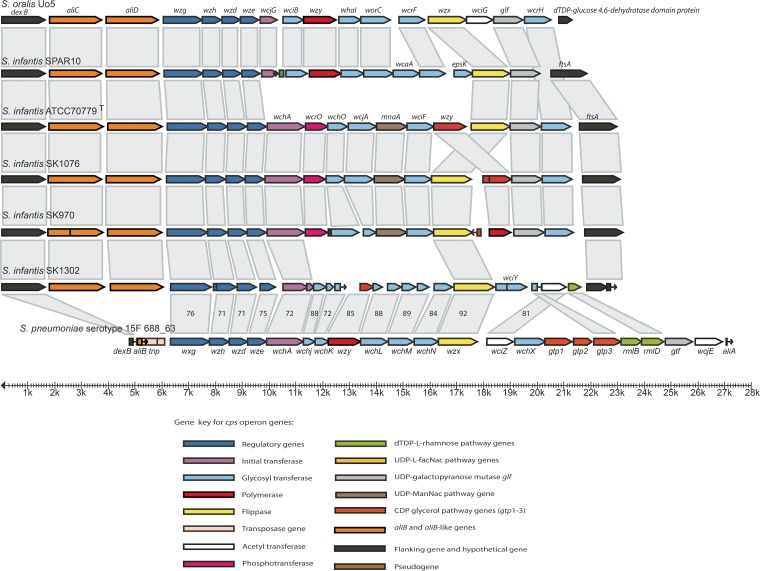
Diagrammatic representation of the capsular biosynthesis loci in strains of *S. infantis*. Gray boxes indicate functional identity as revealed by the annotation, and numbers in selected boxes indicate percentage of nucleotide identity.

The complete *cps* loci of the 16 *S. mitis* strains included up to 26 genes, excluding *dexB* and *aliA* (Fig. 3, 4, and 5)*.* In the remaining five *S. mitis* strains (NCTC 10712, SK321, SK642, SK1080, and B6), the locus between *dexB* and *aliA* consisted of 5,055 to 7,513 bp encoding one or two oligopeptide ABC transporters, AliC and AliD (periplasmic oligopeptide-binding protein OppA), the UDP-galactopyranose mutase Glf (in all but SK642), and the exopolysaccharide biosynthesis transcriptional activator EpsA/Wzg (in NCTC 10712 and SK321) (see Fig. S1 in the supplemental material), suggesting degradation of an originally complete *cps* locus.

An apparent discrepancy was noted for *S. mitis* SK321 between the positive PCR-based demonstration of the regulatory gene *wzg* and the absence of a complete *cps* locus flanked by *dexB* and *aliA*. A search for *cps* genes in other parts of the genome identified a nearly complete *cps*-like locus in another part of the genome flanked by genes encoding recombination helicase AddA (SMSK321_0547) and a hypothetical protein (SMSK321_0548) upstream and a conserved hypothetical protein (SMSK321_0567) and RNase HII (SMSK321_0568) downstream of the *cps* locus.

An analysis extended to all other strains revealed a similar *cps-*like locus at the same genome site in SK137 (SK137_1072 to SK137_1090) in addition to the complete *cps* locus between *dexB* and *aliA*. The *cps*-like loci in the two strains were organized like classical *cps* loci, except that in SK321 only a fragment of the *wzg* gene was present, and in SK137 two of the four regulatory genes, *wzg* and *wzh*, were missing. Theoretically, the missing *wzg* gene in the nonclassical locus of SK321 (*cps2*) may be functionally complemented by the *wzg* gene in the truncated *cps* locus located between *dexB* and *aliA*. Comparison of the classical and nonclassical *cps* loci in SK137 showed no significant homology even between genes that were annotated to carry out similar functions, such as the regulatory genes and the polymerase and flippase genes (see Fig. S2 in the supplemental material). However, the nonclassical *cps-*like loci in SK137 and SK321 were highly similar, except for a duplication of a glycosyl transferase gene (SMSK321_559 and SMSK321_562) in the SK321 *cps*2 locus, and both showed partial similarity to the *S. pneumoniae* serotype 36 *cps* locus (Fig. S2). None of them included *aliB*-like genes.

Among the 10 *S. oralis* subsp. *oralis* genomes analyzed, nine included a full *cps* locus ranging in size between 17,845 and 24,479 bp. In the type strain ATCC 35037/SK23 and in strains ATCC 10557/SK10 and SK144, an acetyltransferase gene was found upstream of the *cps* locus instead of *dexB*. In the four strains Uo5, SK143, SK610, and C104, which showed identical *cps* loci (see below), the *aliA* gene was not present immediately downstream of the *cps* locus. The remaining *S. oralis* strain, SK141, had a 5,888-bp sequence between *dexB* and *aliA* with a structure similar to that of the mentioned *S. mitis* strains with incomplete *cps* loci (Fig. S1).

The five *S. oralis* subsp. *tigurinus* strains, including strain J22, which previously was described as a strain of *S. sanguis* and *S. oralis*, respectively (13, 21), all possessed a full *cps* locus flanked upstream by *dexB* and downstream by *aliA* (Fig. 4B).

The five *S. oralis* subsp. *dentisani* strains (7746, 7747, SK95 [previously “*S. mitis* biovar 2”], F0392 [previously “*S. mitis* biovar 2”], and F0407 [previously taxon 058]) had a complete *cps* locus spanning from 19,743 to 25,018 bp and flanked by *dexB* and *aliA* (Fig. 3F and G)*.* All five *S. infantis* strains had a complete *cps* locus spanning from nucleotides (nt) 18075 to 22149 flanked upstream by *dexB* but not by *aliA* downstream of the *cps* locus (Fig. 5). None of the genomes of the three *S. pseudopneumoniae* strains contained a full *cps* operon (Fig. S1).

In comparison with *S. pneumoniae*, a number of differences in the overall structure of the *cps* operons were observed (Fig. 3 to 5). As described previously, all pneumococcal *cps* operons include one to several transposase genes and several RUP (repeat units in pneumococci) elements (10, 26), which is not the case in any of the other mitis group streptococci examined in this study, except for the two truncated *S. pseudopneumoniae* cps loci (Fig. S1). In addition, immediately downstream from *dexB*, all nonpneumococcus strains had one or two oligopeptide ABC transporter genes (“*aliB*-like”), whereas only fragments were present in some pneumococcal *cps* loci.

The structures of *cps* loci of representative strains were further examined and compared mutually and with *cps* loci of recognized pneumococcal serotypes. The results will be discussed in accordance with the immunochemical results.

### Strains of commensal species with *cps* loci identical to recognized pneumococcal serotypes.

Comparisons of complete sequences and gene contents of *cps* loci of commensal streptococcus strains with those of recognized *S. pneumoniae* serotypes revealed many examples of identity or close similarity (Fig. 3).

*cps* sequences were available for 3 (SK575, SK579, and SK616) of the 10 *S. mitis* strains assigned to *S. pneumoniae* serotype 45 based on serological identity or similarity. In agreement with results of the antigenic analyses, identical *cps* locus structures were found, except for a short fragment of a putative acetyltransferase gene (SPC45_0022) and a putative IS*1381* transposase (SPC45_0023) in the *S. pneumoniae* serotype 45 strain Eddy 72 and *aliC* and *aliD* genes in the *S. mitis* strains (Fig. 3A). Strain SK575 had a gene encoding UDP-galactopyranose mutase Glf (SK575_26) at the end of the locus just upstream of the *aliA* gene. Orthologs of *glf*, but fragmented, were present in the two other *S. mitis* strains, SK579 and SK616 (Fig. 3A). It is not clear if the fragmentation of the reading frames in the two strains is authentic or due to sequencing errors. The genetic analysis offers no explanation for the signs of an extra epitope identified in SK616 relative to *S. pneumoniae* serotype 45 and strains SK575 and SK579.

In accordance with the antigenic analysis, the *cps* locus structure of *S. mitis* strain SK564 was identical to that of *S. pneumoniae* serotype 19C as previously described (26) (Fig. 3B). No other nonpneumococcus strain in the collection showed similarity to this structure. The structure of the cps locus of *S. mitis* SK569 was identical to that of SK564 apart from a truncated UDP-galactopyranose mutase gene (not shown), apparently resulting in loss of the antigenic relationship.

Two strains of *S. mitis*, SK578 and SK1126, and four strains of *S. infantis*, ATCC 70779^T^, SK140, SK970, and SK1076, showed *cps* loci closely related to *S. pneumoniae* serotype 36 (sequence identities of 80 to 88%), but with minor differences that may not influence the expressed polysaccharide (Fig. 3C and 5). While there was extensive sequence homology between regulatory genes, the glycosyltransferases, etc., the flippase and polymerase genes were very distant from those of serotype 36 and were arranged in opposite order in the two *S. mitis* strains (Fig. 3C). Rather, these two genes showed 82% identity to the orthologous genes in *S. pneumoniae* serotype 14 strain Gro Norge. The *cps* loci of *S. infantis* strains SK140, SK970 (not shown), and SK1076 were unique among the five strains in including a gene (*wcwK*/*wefC*) annotated as coding for a capsular polysaccharide phosphotransferase, which had been described as a stealth protein (39) (Fig. 3C).

The *cps* locus of *S. mitis* SK629 was functionally identical to that of the *S. pneumoniae* serotype 33D *cps* locus but with distinct evolutionary histories for three genes. The gene encoding the initial sugar transferase *wcjH* was an ortholog (91% identity) of the gene in *S. pneumoniae* serotypes 39, 43, 47F, and 35F. Interestingly, a 415-nt fragment of the 791-nt glycosyltransferase gene *wciB* following the initial sugar transferase in serotype 39 was also present in SK629. Likewise, the acetyltransferase gene in SK629 showed no homology to the gene in *S. pneumoniae* serotype 33D but was an ortholog of the *wcyO* gene in serotype 39 (66% identity). Finally, the polymerase gene *wzy* showed no significant homology to any pneumococcal *cps* polymerase gene (Fig. 3D).

According to the structure of its *cps* locus, *S. mitis* strain SK667 likely belongs to the *S. pneumoniae* group 18 serovars. As shown in Fig. 3E, it is closely similar to the *cps* loci of serotypes 18F and 18A. Relative to SK667, the serotype 18F locus includes two genes annotated as acetyltransferase genes (SPC18F_0011 and SPC18F_0016). While the former is shared with SK667 (95% identity), the latter is present in SK667 as an intact open reading frame but with nt 175 to 651 missing relative to the intact 1,002-nt gene in serotype 18F. In contrast to all the serogroup 18 *cps* loci, the *cps* locus of SK667 includes three open reading frames (SK667_1776, SK667_1775, and SK667_1772) between *rmlB* and *rmlD* annotated as representing hypothetical proteins. The first of the three is annotated as encoding a conserved protein detected in several strains identified as *S. pneumoniae* from Thailand (for example, WP_050292519.1) (40). The second and largest open reading frame encodes a nuclease-related domain protein with homology to proteins in a strain of *Streptococcus parasanguinis* and in *Streptococcus salivarius* K12. The function of these proteins, if any, in polysaccharide biosynthesis is unknown. In spite of the structural similarity of the SK667 *cps* genes to those of pneumococcus serogroup 18, the SK667 polysaccharide extract did not react with pool Q sera, which react with serotypes 18F, 18A, 18B, and 18C. Thus, an antigenic relationship cannot be confirmed with the available antisera.

The three *S. oralis* subsp. *dentisani* strains SK95, 7747, and F0407 showed *cps* loci identical to that of *S. pneumoniae* serotype 2, apart from a putative cross-wall-targeting SCP domain protein gene present in SK95 and F0407 but absent in strain 7747 and the pneumococcal locus and apart from the two *aliB*-like genes in the *S. oralis* subsp. *dentisani* strains (Fig. 3F). This close genetic similarity in *cps* locus structure is in accordance with complete identity of the polysaccharides of SK95 and *S. pneumoniae* serotype 2 when analyzed with the serotype 2 antiserum. Although the identity was not definitively proven due to the lack of an antiserum against the SK95 polysaccharide, it is likely that the two polysaccharides are identical. The identity of the *cps* locus of the three geographically independent *S. oralis* subsp. *dentisani* strains SK95, F0407, and 7747 out of five analyzed suggest that this is a common serotype in this taxon.

Another *S. oralis* subsp. *dentisani* strain, F0392, was unique among the commensal species but showed 93% nucleotide identity and the same gene content and organization as the *cps* locus of *S. pneumoniae* serotype 5 (Fig. 3G). As only the genome sequence was available, the identity could not be verified by serological analysis.

The *cps* locus of *S. oralis* subsp. *oralis* SK304 was identical to that of *S. pneumoniae* serotype 16A apart from the inverted *rmlD* gene as in all *S. oralis* cps loci with the rhamnose pathway genes (Fig. 3H). The identical arrangement was previously described for *S. oralis* subsp. *tigurinus* strain J22 by Yoshida et al. (21). The *cps* structure demonstrated in *S. oralis* SK304 (Fig. 4B) has not been previously detected.

The serologically detected identity between *S. mitis* strain SK611 and *S. pneumoniae* serotype 40 could not be validated by genetic analysis due to the lack of an available sequence of SK611.

The type strain of *S. oralis* subsp. *tigurinus*, Az_3a, was unique in the collection of commensal streptococci in possessing a *cps* locus virtually identical to that of *S. pneumoniae* serotype 33A, except that the terminal genes annotated as coding for acetyltransferases in both loci are highly dissimilar, although they may have identical functions. Rather, the acetyltransferase gene in *S. oralis* subsp. *tigurinus* strain Az_3a^T^ is an orthologue (88% identity) of the terminal acetyltransferase gene *wzyO* (SPC21_0022) in *S. pneumoniae* serotype 21 strain 546/62. The *cps* locus of Az_3A^T^ is unique among the commensal strains by lacking intact *aliC* or *aliD* genes downstream of *dexB* and by including remnants of an insertion sequence. Immediately upstream of the *aliA* gene, the Az_3a^T^ *cps* locus contains a 401-nt open reading frame with homology to the terminal part of the 1,293-nt IS*1167* transposase gene of *S. pneumoniae* serotype 33A (Fig. 3I).

A summary of the genetic and antigenic identities observed between strains of commensal species and recognized serotypes of *S. pneumoniae* is shown in Table S1.

### Strains of *S. mitis* with limited homology to *S. pneumoniae* serotypes.

Among the remaining *S. mitis* strains for which *cps* locus sequences were available, four groups corresponding to the serologically defined groups smI to smIV were detected (Fig. 4A). Each of these groups of loci showed genes with high sequence identity to recognized *S. pneumoniae* serotypes, while other genes lacked significant homology. Interestingly, the *cps* loci of three of the groups, smI, smII, and smIII, included a gene encoding phosphotransferase DUF 3184 family protein previously demonstrated to have “stealth protein activity” (39).

### Group smI.

In agreement with the immunochemical analysis, three representatives of group I (SK137, SK597, SK608) showed identical *cps* structures, with the exception that the *cps* locus of SK597 encoded both versions of the oligopeptide ABC transporters AliC and AliD, while the two other strains possessed the *aliD* gene only (see below). The serological analysis of SK608 suggested a missing epitope in the polysaccharide relative to that observed for SK137 and SK697. However, the gene content of the *cps* operons of the three strains does not provide an explanation for this possible difference in epitope structure. The glycosyltransferase genes are highly similar in the three strains. Apart from the *wciB* gene downstream of the initial transferase, all glycosyltransferase genes lack significant homologies to pneumococcal *cps* loci. Noticeably, the operon also encodes an LPTXG cell wall anchor protein (Fig. 4A).

### Group smII.

In agreement with the serological analysis, the *cps* locus of the type strain of *S. mitis* NCTC 12261/SK142 was unique in the strain collection (Fig. 4A). Although the overall structure showed no significant homology to any of the pneumococcal *cps* loci, several genes had orthologs both in *cps* loci of *S. pneumoniae* and several other species. In addition to genes encoding the four regulatory proteins and the initial sugar transferase WchA gene (SM12261_0992), the rhamnosyltransferase WchF gene (SM12261_0993), a glycosyltransferase gene (SM12261_0994), the flippase Wzx gene (SM12261_0999), and the four rhamnose pathway genes *rmlA* to -*D* are shared with many serotypes of *S. pneumoniae*. Other genes were rare or absent among pneumococci, such as genes encoding the putative acetyltransferase (SM12261_0995) found only in serotypes 7F, 7A, 22F, and 22A, the two putative glycosyltransferase genes SK12261_0996 and SK12261_0997 found in none of the pneumococcal serotypes, the polymerase Wzy gene (SM12261_0998) in serotypes 13, 35F, 35B, 36, and 47F and all members of serogroup 18, the putative glycosyltransferase gene SM12261_1001 in serotypes 20 and 21, and the putative galacto-furanosyltransferase gene (SM12261_1002) in serotypes 10F, 10C, 29, 35B, 36, and 43.

### Group smIII.

Comparison of the *cps* loci of strains SK271 and SK1073 among the four *S. mitis* strains assigned to this serogroup showed an identical structure, in agreement with the observed serological identity. The structure of the *cps* locus is shown in Fig. 4A. Apart from the four rhamnose pathway genes *rmlA* to -*D*, no overall identity or close similarity to any of the *S. pneumoniae* cps loci was observed. The conserved hypothetical protein encoded by gene SK271_1556 is 63% identical to the WcwD protein encoded by the *cps* locus of *S. pneumoniae* serotype 7F and has orthologs in many other *Streptococcus* species. Genes that were not represented by orthologs in any of the *cps* loci of recognized serotypes of *S. pneumoniae* were encoding an acetyltransferase (“LbH_MAT_like,” SK271_1557) previously demonstrated in so-called atypical pneumococci (40) and one glycosyltransferase (SK271_1555) with only 33% amino acid sequence identity to glycosyltransferases in *S. pneumoniae.*

### Group smIV.

The single *S. mitis* strain, SK637, assigned to this group had a *cps* locus spanning 17,161 nt (Fig. 4A). Although the overall structure was distinct, all genes had orthologs in *S. pneumoniae* cps loci. A span of three glycosyltransferase genes, SK637_1569 to -67 had orthologs in the *cps* locus of *S. pneumoniae* serotype 39.

### Genetic analysis of *cps* loci in *S. oralis* subspecies *oralis*, *dentisani*, and *tigurinus* and in *S. infantis*.

The *cps* locus of the *S. oralis* subsp. *dentisani* strain 7746^T^ was not identical to any *S. pneumoniae* cps locus but showed partial similarity in gene content to that of *S. pneumoniae* serotype 33F. In addition to the four regulatory genes, orthologs of serotype 33F *cps* genes included those encoding the initial sugar transferase (WchA), the flippase (Wzx), a putative acetyltransferase (WciG), and the UDP-galactopyranose mutase Glf of serotype 33F. The final pseudogene of another putative acetyltransferase (WcjE), present in serotype 33F, is not present in the 7746^T^ *cps* operon (Fig. 4B). The glycosyl transferase gene *wciN* and the LicD protein phosphotransferase gene *wcrO* were shared with the *cps* locus of serotype 33C.

Surface polysaccharides encoded by the *cps* locus of *S. oralis* have been studied both genetically and structurally by Cisar and coworkers (13, 17, 20, 21), who have been using the term coaggregation receptor polysaccharides (CRPs) according to their demonstrated function and specificity in interspecies coaggregation processes during biofilm formation on tooth surfaces. According to the designations used by Cisar and coworkers (41), the type strain of *S. oralis* ATCC 35037 and *S. oralis* strain ATCC 10557 both had a *cps* locus corresponding to the type 3G coaggregation receptor polysaccharide. *S. oralis* subsp. *tigurinus* strain SK313 had a *cps* locus identical to that of type 2G represented by *S. oralis* subsp. *tigurinus* strain J22 (previously named *S. sanguis* and *S. oralis*, respectively) (Fig. 4B). The *cps* locus type 4Gn represented by *S. oralis* strain C104 was found also in strains SK143, Uo5, and SK610. Type 1Gn found in *S. oralis* strain 34 was unique among the strains examined in this study. None of these *cps* locus types showed homology in gene structure to that of recognized *S. pneumoniae* serotypes. An exception was *S. oralis* SK144 (structural type 5Gn). The difference between the *cps* locus of this strain and that of SK143 (4Gn) was the acetyltransferase gene missing in SK144 (Fig. 4B). An ortholog of this acetyltransferase gene is found in *S. pneumoniae* serotypes 10C and 10F, with which both SK143 and SK144 share a significant part of the *cps* locus genes (Fig. 4B). One additional type, not previously reported, was demonstrated by our genetic analysis. As described above, *S. oralis* strain SK304 had a *cps* locus identical to that of *S. pneumoniae* serotype 16A (Fig. 3H and 4B).

The *cps* loci of two *S. oralis* subsp. *tigurinus* strains, SK255 and SK1074, each were unique in the collection. Besides by its gene content, the SK255 *cps* locus included an integrase core protein gene between the final acetyltransferase and the flanking *aliA* (Fig. 4B). The other unique *S. oralis* subsp. *tigurinus* strain, SK1074 (Fig. 4B), showed from 76 to 95% nucleotide sequence identity with the genes encoding the four regulatory proteins, the initial sugar transferase, the putative rhamnosyl transferase WchF, and the four rhamnose pathway proteins RmlA to -D in the *cps* loci of *S. pneumoniae* serotypes 2 and 7F. Likewise, the flippase gene *wzx* and the glycosyltransferase gene immediately upstream shared 66 to 72% nucleotide sequence identity with genes in the *S. pneumoniae* serotype 47A *cps* locus. All remaining seven genes in the central part of the *cps* locus of SK1074 lacked homologs among available sequences from *Streptococcaceae*, although several were annotated as encoding glycosyltransferases and an acetyltransferase. Surprisingly, no gene showed homology to any available sequence of a polymerase. Finally, like two other strains of *S. oralis* subsp. *tigurinus* (Az_3a^T^ and SK255), the *cps* of SK1074 included a fragment of a transposase gene.

The *cps* locus of *S. infantis* SPAR10, flanked by *dexB* and *ftsA*, was closely similar to that of *S. oralis* strains Uo5, C104, SK143, and SK610, except for two additional glycosyl transferase genes (*wcaA* and *epsK*) in SPAR10 and an acetyltransferase (*wciG*) in the *S. oralis* strains (Fig. 4B and 5). In *S. infantis* SK1302, the locus showed partial identity in structure and sequence to that of *S. pneumoniae* serotype 15F. The exceptions are the genes downstream of the flippase gene *wzx* (Fig. 5). As strain SK1302 was lost, the identity of the capsular structure could not be definitively proven by serological analysis.

### Genes unique to *cps* loci of commensal streptococci.

The *cps* loci of many commensal streptococci include one or two genes encoding periplasmic oligopeptide-binding protein, so-called “AliB-like” or “AmiA” proteins. According to Park et al. (42), the genes may be termed *aliC* and *aliD.* A phylogenetic analysis of the genes extracted from all *cps* loci examined in this study plus reference sequences from the report of Park et al. (42) allowed us to assign names to the individual genes. According to the tree shown in Fig. S3a in the supplemental material, two major clades, each containing one of the two reference sequences *aliD* and *aliC*, were observed. Within each clade, separate clusters reflecting the overall phylogeny of the individual species are seen. These clusters, therefore, constitute allelic versions of the same gene (i.e., *aliD* and *aliC*, respectively). The gene *aliD* was present in all complete *cps* loci of commensal streptococci and in strains of the three *S. pneumoniae* serotypes 25A, 25F, and 38. As demonstrated by Bentley et al. (10), the *cps* loci of these three *S. pneumoniae* serotypes include an almost complete sequence (1,917 and 1,959 nt) of *aliD* but with three premature stop codons created by two minor sequence deletions. The phylogenetic analysis presented in Fig. S3a shows that the additional *aliB-*like genes present in strains of *S*. infantis and *S. oralis* subspecies *oralis*, *dentisani*, and *tigurinus* are *aliC*. This gene is absent in *S. mitis* strains, with the exception of SK597 and SK629 (Fig. 3 to 5). The genes in Fig. 3 to 5 (Fig. S1 and S2) are named according to this phylogenetic analysis. In all *S. pneumoniae* cps loci other than serotypes 25A, 25F, and 38, a pseudogene consisting of the first 153 to 174 nt of the 1,959 nt in *S. mitis* aliD was present*.* As previously demonstrated by Hathaway et al. (43), the truncated *cps* region of nonencapsulated pneumococci contains one or two *aliB*-like genes. The clustering of these genes in the tree (Fig. S3a) shows that they are orthologs of *aliC* and *aliD* organized as in strains of commensal streptococci.

Table S5 in the supplemental material provides a summary of *cps*-locus encoded proteins in commensal streptococci that do not have significant matches among *cps* locus-encoded *S. pneumoniae* proteins (above 50% amino acid sequence identity over >30% of the length).

### Phylogenetic analysis of selected *cps* locus genes.

A phylogenetic analysis of *wzy* gene sequences from all *S. pneumoniae* serotypes and commensal streptococci with the available information on the linkage specificity of the encoded polymerase is presented in Fig. S3b. Combined with the significant sequence diversity among *S. pneumoniae* serotypes, the identities of many pneumococcal genes with *wzy* genes of several commensal streptococci are in agreement with our observation that the diversity of *S. pneumoniae* cps loci and capsular serotypes emerged by acquisition of genes from other species (26).

### Other proteins encoded by *cps* locus genes.

The *cps* loci of three *S. mitis* strains belonging to serogroup I, SK137, SK597, and SK608, included a gene encoding a putative cell-wall-anchored protein with an LPXTG motif at the N terminus (Fig. 4A). The encoded 985-aa, 979-aa, and 999-aa proteins showed 84 to 92% mutual amino acid identity and belong to the G5 superfamily. Bentley et al. (10) identified a putative surface-anchored protein gene at the end of the *cps* locus of *S. pneumoniae* serotype 14. However, the three *S. mitis* proteins showed no homology to the *S. pneumoniae* protein or to any other protein in the NCBI database, and their function in the context of capsular polysaccharide synthesis, if any, remains obscure.

The *cps* locus of two of the five strains of *S. oralis* subsp. *dentisani*, SK95 and F0407, included a gene encoding a protein with a putative choline-binding, cross-wall-targeting lipoprotein signal (SCP domain extracellular protein) between the two periplasmic oligopeptide-binding protein genes *aliC* and *aliD* (Fig. 3F). BLASTP screening of the NCBI nonredundant protein database shows that homologs are present in many commensal streptococci and in an unpublished *S. pneumoniae* strain, 2080767 II, isolated from blood (SAMEA2382970).

### Annotation of genes of the *S. mitis* SK137 *cps* locus.

We previously determined the structure of the SK137 capsular polysaccharide (16) (see Fig. S4 in the supplemental material). This allows us to propose the function of some of the proteins encoded by the genes in the *cps* operon of this strain and thus annotate most of the genes in the capsular biosynthetic locus (see Table S3 in the supplemental material). The gene downstream of *dexB* encodes an AliD periplasmic regulatory protein (SMSK13_0341). This gene is similar to *aliA* immediately downstream of the *cps* locus. It has been suggested that this group of proteins are involved in substrate recognition (44, 45) and may not participate directly in the polysaccharide synthesis. Seven other genes of the SK137 *cps* locus are common to the Wzy-dependent capsular polysaccharide biosynthesis pathway. They encode enzymes/proteins involved in the process of regulation and cell wall linkage (genes 0342, transcriptional regulator; 0343, tyrosine-protein phosphatase; 0344, chain length determinant protein; and 0345, tyrosine-protein kinase), oligosaccharide chain elongation (0348 plus 0349, Wzy repeat unit polymerase), and transfer of repeat units across the cell wall (0355, Wzx flippase) (11). Based on alignment of related protein sequences and a search among published polysaccharide synthesis pathways, the functions of some of the remaining enzymes encoded by the SK137 *cps* locus genes are suggested (Table S2). One gene (0356) encodes a mutase (Glf) that catalyzes the transformation of galactopyranose to galatofuranose, a monosaccharide appearing twice in the polysaccharide structure (residues I and VII [Fig. S4]) of SK137. The suggested specificities of the six transferases are listed in Table S3 and see Fig. S4. The process is started by an initial transferase (Fig. S4, bond 1, gene 0346) that links glucosyl-1-phosphate from UDP-glucose to a lipid carrier (11, 46). The second monosaccharide next to the glucose moiety is Gal*f*. The linkage (bond 2a; see Fig. S5 in the supplemental material) is established by an enzyme (0347) similar to the product of the transferase *wciB* gene (76 to 84% identity) present in various *S. pneumoniae* serotypes, which like SK137, have the d-Gal*f*-(1→3)-β-d-Glc*p* unit (11, 27, 47) (Table S2). The third and fourth sugars are Gal*p* moieties attached by β(1–6) glycosidic linkages (bonds 2b and 2c, Fig. S5). The transferases (genes 0350 and 0351, core-2/I-branching enzyme [pfam02485]) catalyzing these two bonds are somewhat related (40 to 45% identity) to the products of the *wcrM* and *wcrG* genes in *S. pneumoniae* serotypes 29 and 35B and in serotypes 10A and 39, respectively (11, 27, 48) (Table S3). The five mentioned pneumococcal polysaccharides contain a glycosylic linkage shared with SK137. However, the repeat units differ as one of the two Gal molecules in the disaccharides from pneumococci is acetylated. The fifth sugar, Glc*p*-1-P (V, step 2 days), is attached by an α-glucose 1-phosphotransferase (0354), similar to the product of the *wcrK* gene (45% identity) present in *S. pneumoniae* serotype 7B (11, 27). The next bond (2e, Fig. S5) is an α(1–6) glycosylic linkage that may be formed by the action of an α-glycosyl transferase. This step is, however, uncertain because the putative gene (0352) is not closely related to any other gene encoding an enzyme with a known function. The last sugar, Gal*f* (VII; Fig. S5), is transferred by a galactofuranosyl transferase (0353) related to the product of the *wcrH* gene (38% identity), which forms the same, although inverted, linkages (i.e., β instead of α) in pneumococcal serotype 10F polysaccharide (11), and *wefE* (38% identity) in *S. oralis* (49). The polymerase (0348 plus 0349, one gap) connects the repeat units by catalyzing the formation of the d-Glc*p*-(1→6)-β-d-Gal*f* linkages in the final SK137 capsular polysaccharide.

### *cps* loci in other commensal *Streptococcus* species.

Using a BLASTp search of selected representatives of genomes of other species of streptococci commensal to the upper respiratory tract and oral cavity, we identified complete *cps* loci in all examined strains of *Streptococcus anginosus*, *Streptococcus intermedius*, *Streptococcus constellatus*, *Streptococcus cristatus*, *Streptococcus parasanguinis*, *Streptococcus australis*, and *Streptococcus gordonii* (see Table S4 in the supplemental material), with the reservation that some were distributed on more than one contig. All *cps* loci in these species were located in the genomes immediately downstream of a gene encoding an anaerobic ribonucleoside-triphosphate reductase-activating protein. All contained the four regulatory genes *wzg*, *wzh*, *wzd*, and *wze*, except for *S. australis* ATCC 700641, from which *wze* was missing. In several of the anginosus group streptococci, transposase genes or gene fragments were present as in *cps* loci of *S. pneumoniae*. In none of these strains did the *cps* locus include *aliB*-like genes.

## DISCUSSION

Expression of a capsular polysaccharide is considered a hallmark of most invasive species of bacteria. In invasive strains of *S. pneumoniae*, the capsule is among the principal virulence factors, as demonstrated by results of *in vitro* experiments, experimental infections, and the success of the current conjugate vaccines based on selected serotypes of capsular polysaccharides. Consequently, it was previously assumed that capsule production distinguishes *S. pneumoniae* from closely related commensals of the mitis group streptococci. The findings of this study effectively disprove this assumption. Our genetic analyses demonstrated complete *cps* loci in 74% of 66 random *S. mitis* strains, in all but one of 20 *S. oralis* strains, including the subspecies *oralis*, *tigurinus*, and *dentisani*, and in all six *S. infantis* strains*.* Searches of complete genome sequences in GenBank further revealed complete *cps* loci in all examined strains of the mitis group species *Streptococcus cristatus*, *Streptococcus parasanguinis*, *Streptococcus australis*, and *Streptococcus gordonii* and in the more distantly related anginosus group species *Streptococcus anginosus*, *Streptococcus intermedius*, and *Streptococcus constellatus* (Table S4). The antigenic analyses confirm that the capsular polysaccharides are expressed. The only exception appears to be *S. pseudopneumoniae*, which had a significantly truncated *cps* locus similar to that of occasional *S. mitis* and *S. oralis* strains (Fig. S1). The high prevalence of intact *cps* loci in *S. mitis* is at odds with the observation recently reported by Yang et al. (23) that none of 12 *S. mitis* strains examined by them contained a *cps*/*rps* operon.

The location of the *cps* locus in the genomes reflects, to a large degree, the extensive synteny of genomes of mitis group *Streptococcus* species. Like in *S. pneumoniae*, the *cps* locus was flanked by *dexB* and *aliA* in all strains of *S. mitis*, *S. oralis* subsp. *tigurinus*, and *S. oralis* subsp. *dentisani*. In *S. infantis* strains, the flanking gene downstream of the *cps* locus was not *aliA* but *ftsA*. Surprisingly, *S. oralis* subsp. *oralis* strains showed different patterns. While strains SK304, C104, and 34 were identical to *S. pneumoniae*, *S. mitis*, *S. oralis* subsp. *tigurinus*, and *S. oralis* subsp. *dentisani*, other strains of *S. oralis* subsp. *oralis* lacked either the downstream *aliA* or upstream *dexB* gene (Fig. 4B; see Table S1 in the supplemental material). Furthermore, in view of the close genetic relationship of *S. pseudopneumoniae* to *S. pneumoniae* and *S. mitis*, it is surprising that *aliA* is not found downstream of its truncated *cps* locus, in contrast to that of nonencapsulated strains of *S. pneumoniae* and *S. mitis* (Fig. S1).

The *cps* loci of pneumococci are among the genome areas most frequently affected by recombination events (50). Horizontal transfer of *cps* genes between strains is facilitated by the several transposase genes and RUP elements present in the *cps* loci of all pneumococcal serotypes (10). As part of the many genetic traits that contribute to the genomic stability of *S. mitis*, contrasting with the genomic plasticity of *S. pneumoniae*, we previously demonstrated that transposases and RUP elements are lacking in *cps* loci of *S. mitis* (26). This is confirmed by this study for *S. mitis* and furthermore demonstrated for other commensal species, with the exception of *cps* loci of strains of *S. oralis* subsp. *tigurinus*, S. pseudopneumoniae, and species of the more distant anginosus group, which included transposase genes (Fig. 3 and 4; Fig. S1).

We previously demonstrated that the structural polymorphism of capsular polysaccharides in *S. pneumoniae* evolved by import of relevant genes from a range of commensal *Streptococcus* species (26). Therefore, it was not surprising to find strains of commensal streptococci with *cps* loci identical or nearly identical in gene structure to those of recognized pneumococcus serotypes (Table S1; Fig. 3 to 5). A total of 26% of the detected *S. mitis* capsules were structurally identical to pneumococcal serotypes. However, the patterns of nucleotide sequence identities over the range of the *cps* locus clearly demonstrate that the *cps* gene import by *S. pneumoniae* does not occur *in toto* but as blocs of genes resulting in a mosaic of genes of different origins. This is most clearly demonstrated in the range of identities between genes of *S. pneumoniae* serotype 5 (strain Ambrose) and *S. oralis* subsp. *dentisani* strain F0392 (Fig. 3G), between *S. pneumoniae* serotype 33D (strain CSF 79) and *S. mitis* SK629 (Fig. 3D), and between *S. pneumoniae* serotype 16A (strain R105) and *S. oralis* SK304 (Fig. 3H). It is conceivable that the pneumococcal import of *cps* locus genes is a still ongoing process that will result in novel capsular polysaccharide structures in *S. pneumoniae*, some of which may be identical or similar to structures that presently are unique to commensal species. Like those of *S. pneumoniae*, the capsular polysaccharides of commensal species, in particular *S. mitis*, showed a significant degree of structural diversity as indicated by the antigenic and genetic evidence. In addition to eight structures identical to recognized pneumococcal serotypes and four structures unique to *S. mitis* strains, 43% of the examined strains with a putative complete *cps* locus did not react in any of the available antisera. Apart from the potentially different mosaics of genes that may lead to different structures of polysaccharides, several genes in the *cps* loci of commensal species annotated as glycosyl transferases lacked homologs in the current *S. pneumoniae* serotypes (Table S5). Although the exact transferase activities of these enzymes are yet unknown, it is possible that they can expand the structural diversity of capsular polysaccharides if imported by pneumococci.

The demonstrated antigenic identity of at least eight capsular polysaccharides from commensal streptococci with recognized serotypes of *S. pneumoniae* (serotypes 2, 5, 16A, 18F, 19C, 33A, 33D, 36, and 45) raises important questions concerning the consequences for host-parasite relationships and the potential impact on pneumococcal infections. Does colonization with such commensal strains influence the prevalence of cross-reacting pneumococcal serotypes, induce immunity, or increase infection susceptibility to them? As the necessary comprehensive epidemiological data are not available, the questions can be approached only from a theoretical point of view. One of the capsules detected in the examined collection of commensal streptococci (i.e., *S. oralis* subsp. *dentisani* strain F0392) was identical to *S. pneumoniae* serotype 5, which is among the frequent causes of pneumococcal infection and is included in the current 13-valent conjugate vaccine (51). Therefore, inadvertent elimination of members of the commensal microbiota of the upper respiratory tract by the pneumococcus vaccination is of potential concern. Conversely, there is increasing evidence that commensal bacterial species induce immunological tolerance at the mucosal level but not in the systemic compartment of the immune system, thus facilitating their harmonious coexistence with the host as long as they remain in their natural habitat (52). Therefore, the demonstrated cross-reacting commensal streptococci are neither likely to induce protection nor provide enhanced susceptibility to pneumococcal infection by the mechanisms hypothetically related to the production of an IgA1 protease (53, 54).

In pneumococcal infections, the capsular polysaccharide confers a strong antiphagocytic activity on the bacteria, at least partly by reducing the complement deposition on the bacterial surface (55). *In vitro* studies show that serotypes that are resistant to neutrophil-mediated killing tend to be more heavily encapsulated (56). While the pneumococcal capsules are estimated to be approximately 200 to 400 nm thick (57), information on the size of capsules of commensal *Streptococcus* species is largely lacking. Yurchak and Austrian (37) reported that the capsular reaction test (“capsular quellung”) is not optimal for detection of surface polysaccharides in nonpneumococal streptococci due to the relatively small amounts of capsular polysaccharide, but the identity of the strains is not clear. It is generally assumed that capsular polysaccharides in *S. pneumoniae* cover other antigens located on the surface of the bacterial cell wall, as we showed for the pneumococcal group O antigen (C-polysaccharide) (57). Our observation that the formalin-treated cells of commensal streptococci aggregated by a proteinase K-sensitive mechanism suggests that the capsular polysaccharide does not cover surface-exposed proteins as in pneumococci. An additional important difference may be that capsule expression in *S. pneumoniae* is under regulation by a mechanism mediated by a type I restriction modification system (SpnD39III) (58), which is lacking in commensal *Streptococcus* species (Fig. S5).

In addition to the capsule, commensal streptococci may produce two other extracellular polysaccharides. One is the cell wall polysaccharide analogous to the so-called C-polysaccharide or Lancefield group O antigen in pneumococci (16). Species such as *S. oralis* subsp. *oralis*, *S. sanguinis*, and *S. gordonii*, in addition, produce an extracellular glucan synthesized by a surface-associated glycosyltransferase (59) by a mechanism similar to that of the serotype 3 capsular polysaccharide in pneumococci. In contrast to capsules (60), these extracellular polysaccharides are not covalently linked to the call wall. In oral streptococci, the extracellular glucan is known to play a role as intercellular matrix in the biofilms formed by these bacteria on, for example, tooth surfaces (61). The molecular interplay between these polysaccharides and the capsular polysaccharide, which is actively exported to the surface, is yet unknown.

Pioneering work by Cisar and his coworkers demonstrated that surface polysaccharides synthesized by the Wzy/Wzx pathway in strains of *S. oralis*, *S. gordonii*, and *S. sanguinis* mediate coaggregation between members of the biofilm formed on tooth surfaces (17, 23). These polysaccharides have been referred to as coaggregation receptor polysaccharides (CRPs), but are the equivalent of capsular polysaccharides of pneumococci and the commensal species examined in this study. Although the coaggregation mechanism has been mapped only in strains of *S. oralis*, *S. gordonii*, and *S. sanguinis*, it is likely that the polysaccharides demonstrated in this study for many additional species, including *S. mitis*, have similar functions. In addition, capsulation may protect bacteria from attack by bacteriophages as demonstrated for pneumococci (62). However, it is still unknown to what extent expression of a capsular polysaccharide may contribute to the survival of commensal streptococci that gain access to the bloodstream and thus may play a role in the pathogenesis of subacute bacterial endocarditis.

Interestingly, the *cps* loci of all examined strains of *S. mitis*, *S. oralis* subspecies *oralis*, *tigurinus*, and *dentisani*, and *S. infantis* include one or two genes encoding an AliB-like protein. These proteins belong to a family of paralogous membrane-bound lipoproteins, AmiA, AliA, and AliB, that participate in oligopeptide transport in *S. pneumoniae*. The gene encoding AliA is found in all pneumococci and strains of *S. mitis* and *S. oralis* subspecies *dentisani* and *tigurinus*, as well as in some strains of *S. oralis* immediately downstream of the *cps* locus but with no known function in capsular polysaccharide biosynthesis. One or two alleles of the *aliB*-like genes, *aliC* and *aliD*, were found in all complete as well as truncated *cps* loci of *S. mitis*, *S. oralis* subsp. *dentisani*, *S. oralis* subsp. *tigurinus*, and *S. infantis* immediately downstream of *dexB* (Fig. 3 to 5; Fig. S1) but not in *S. anginosus*, *S. intermedius*, *S. constellatus*, *S. cristatus*, *S. parasanguinis*, *S. australis*, *S. sanguinis*, and *S. gordonii*. Orthologous genes at the start of the *cps* locus were previously demonstrated in nonencapsulated strains of *S. pneumoniae* (40, 42, 44). Remarkably, the *cps* locus of none of the encapsulated pneumococci includes functional *aliB*-like genes although there are traces of their prior existence in the form of small fragments in most serotypes and almost full-length pseudogenes in strains of serotypes 25A, 25F, and 38 (10, 43) and in *S. pseudopneumoniae* (Fig. S1). The pressure that eliminated the genes in encapsulated pneumococci but not in their noninvasive counterparts (i.e., nonencapsulated pneumococci and the most closely related species *S. mitis*, *S. oralis*, and *S. infantis*) is unknown, but might be related to their parallel adaptation to either pathogenic or mutualistic lifestyles (26). Claverys et al. (45) and Hathaway et al. (44) reported that AliA and AliB-like proteins are involved in sensing environmental conditions by their ability to detect and respond to foreign bacterial peptide fragments in their environment. In this context, their proximity to capsular biosynthesis genes and their potential regulatory effects in commensal streptococci are of obvious interest.

In conclusion, capsular polysaccharides synthesized by the Wzy/Wzx pathway are generally expressed by commensal streptococci associated with humans. The level of sequence identities of *cps* locus genes confirms that the structural polymorphism of capsular polysaccharides in *S. pneumoniae* evolved by import of *cps* fragments from commensal *Streptococcus* species, resulting in a mosaic of genes of different origins. Like in *S. pneumoniae*, a significant structural diversity of capsular polysaccharides was demonstrated in commensal species, in particular in *S. mitis*. The demonstrated antigenic identity of many capsular polysaccharides expressed by commensal streptococci and *S. pneumoniae* raises important questions concerning the consequences for host-parasite relationships both for the commensals and for the pathogen *S. pneumoniae*.

## MATERIALS AND METHODS

### Bacterial strains and growth conditions.

A total of 201 mitis and anginosus group streptococci were examined: *S. infantis* (*n* = 6), *S. mitis* (*n* = 66), *S. oralis* subsp. *oralis* (*n* = 11), *S. oralis* subsp. *tigurinus* (*n* = 5), *S. oralis* subsp. *dentisani* (*n* = 5) *S. pseudopneumoniae* (*n* = 3), *Streptococcus* sp. strain ATCC 6249, and encapsulated strains of *S. pneumoniae* (*n* = 90, i.e., one strain each of the recognized 97 pneumococcal serotypes, except for the seven recently described serotypes 6C to -H and 11E), *S. cristatus* (*n* = 3), *S. parasanguinis* (*n* = 2), *S. australis* (*n* = 1), *S. gordonii* (*n* = 2), *S. anginosus* (*n* = 2), *S. constellatus* (*n* = 2), and *S. intermedius* (*n* = 1). Among the nonpneumococcus strains, 27 strains were represented only by DNA sequence data downloaded from the NCBI database. The remaining strains were from our own or national bacterial culture collections. The identity of the strains was according to the most recent taxonomic updates based on core genome analyses (63). The streptococcus strains were cultured on either 5% blood agar plates (Statens Serum Institut, Copenhagen, Denmark) or in Todd-Hewitt broth (CM189; Oxoid) overnight at 35°C in a 5% CO_2_ incubator.

### Genetic analyses.

The structures of the capsular polysaccharide biosynthesis locus, *cps*, of 90 capsular serotypes of *S. pneumoniae* reported by Bentley et al. (10) and 52 commensal strains extracted from available complete or draft genome sequences were examined and compared in a Sybil database constructed as described previously (64) and established as part of this study (accessible at http://sybil-clovr.igs.umaryland.edu/sybil/Kilian_CPS_loci). The latter included *S. mitis* (*n* = 22), *S. pseudopneumoniae* (*n* = 3), *S. oralis* subsp. *oralis* (*n* = 10), *S. oralis* subsp. *tigurinus* (*n* = 5), *S. oralis* subsp. *dentisani* (*n* = 5), *S. infantis* (*n* = 6), and *Streptococcus* sp. strain ATCC 6249 (*n* = 1). A complete list of these strains and accession numbers for the sequences is shown in Table S1. Nucleotide and protein sequence BLAST analyses were performed at the NCBI database. Illustrations generated in Sybil were manually edited in Adobe Illustrator.

Cluster analysis of selected *cps* genes were carried out in MEGA version 6.06 (65) using the Minimum Evolution algorithm and bootstrap analysis with 500 replicates.

### PCR detection of *cps* locus genes.

The presence of the regulatory gene *wzg* of the *cps* operon was examined in 66 *S. mitis* strains by PCR using two sets of primers: wzg-1-for (AATGCRRCITCIAAYTAYTCARTATTC) combined with wzg-1-rev (CCRTARGTRTCAATICCRCTIAYATA) and wzg-2-for (AGTGTIAYRGSICCRACWGRIACIRATAAKGA) combined with wzg-2-rev (TCIATCAWYTTCAARAAIGARGTRAARTTCAAICG), where “I” stands for deoxyinosine. The amplicons of 401 and 575 bp, respectively, generated by the wzg-1 and wzg-2 primer sets were detected by agarose gel electrophoresis. For the PCR, we used PuReTaq Ready-to-Go PCR beads (GE Healthcare, United Kingdom) in a 25-μl reaction mixture containing 1 ng genomic DNA and 50 pmol of each primer. A thermocycling program of 96°C for 1 min, 30 cycles of 96°C for 30 s, 55°C for 30 s, and 72°C for 1 min followed by an extension at 72°C for 5 min was used.

### Antisera.

Antisera were raised against the following 12 streptococcus strains: the type strains of *S. mitis* (SK142 = NCTC 12261) and *S. oralis* (SK23 = ATCC 35037) and 10 additional *S. mitis* strains, SK137, SK271, SK564, SK569, SK575, SK597, SK608, SK611, SK637, and SK1124, selected based on positive PCRs for *cps* locus genes suggesting the potential for surface polysaccharide expression. Briefly, bacterial cells stabilized in 1% formaldehyde were collected by centrifugation (3,000 × *g*, 30 min), washed in phosphate-buffered saline (PBS), and treated with 10 µg proteinase K per ml concentrated cell suspension for 1 h at room temperature (66). Bacterial aggregations were hereby dissolved, or extra proteinase K was added. After the treatment, the enzyme and peptides of digested proteins were removed by washing the cells twice in PBS. White New Zealand female rabbits (2 kg) were immunized by intravenous injections of 1 ml the stabilized proteinase K-treated whole-cell vaccine as described previously (67) under an official permit and in agreement with the national guidelines for animal research. Titers of the prepared antisera were individually examined by double immunodiffusion (Fig. 1). Sera from weekly bleedings of two rabbits immunized with the same antigen and with an agglutination titer equal to 16 or higher were pooled. Diagnostic pneumococcal antisera (pools, group, and type sera) were obtained from Statens Serum Institut, Copenhagen (68).

### Preparation of streptococcal polysaccharide extracts for immunoprecipitation.

Bacterial cells were harvested from 40-ml overnight broth cultures by centrifugation (3,000 × *g*, 30 min) and lysed and treated as follows. (i) Nonpneumococcal cells were suspended in 1-ml lysis buffer (0.1 M NaCl, 0.05 M HEPES, 1 mM CaCl_2_, 1 mM MgCl_2_ [pH 7.5]) containing 100 U of mutanolysin (Sigma) and 1 mg lysozyme (Sigma) and incubated at 37°C for several hours until more than 95% of the cells were digested as evaluated by Gram staining. (ii) Pneumococcal cells were suspended in 1 ml 0.1% sodium deoxycholate in PBS. This lysis buffer activates the autolysin and induces complete lysis of the pneumococcal cells. Cell debris was removed from the bacterial extracts by centrifugation (10,000 × *g*, 30 min), and proteins in the supernatants were digested by adding proteinase K (10 µg/ml) for 2 h at 50°C. The protease activity was finally blocked by adding 15 µl stock solution of the protease inhibitor phenylmethylsulfonyl fluoride (PMSF [with 17.4 mg/ml isopropanol]) per ml of extract to a final concentration of 1.5 mM. A 0.01% solution (1 mg per 10 ml of saline) of purified pneumococcal C-polysaccharide (Statens Serum Institut) was used as a control.

### Examination of crude bacterial antigen extracts by immunoprecipitation.

Crude extracts of the bacterial strains used for immunization of rabbits were prepared for evaluation of the specificity of the raised sera. Mutanolysin-lysozyme extracts were prepared from live bacterial cells as described above, except that proteinase K was omitted initially. The crude extracts contained mixtures of polysaccharide and protein antigens and were treated as follows. (i) Each extract was kept without further treatment. (ii) Extract was mixed with proteinase K stock solution (100 µg/ml, 2 h, 50°C). (iii) Extract was mixed (10:1) with freshly prepared 0.1 M sodium metaperiodate solution in 0.5 M acetate buffer (pH 5.0). After incubation of the sample on ice for 1 h, the reaction was stopped by adding 25 µl of polyethylene glycol solution (30% PEG 8000 in 0.3 M NaHCO_3_-NaOH [pH 8.0]). (iv) Extract was mixed (10:1) with 0.5 M acetate buffer (pH 5.0) without periodate (negative control). The four different specimens (i to iv) made from each extract were then compared by double immunodiffusion against the homologous antiserum as shown in Fig. 1. Double immunodiffusion in agarose was used for the examination of reaction between streptococcal polysaccharide extracts (antigens) and the different rabbit antisera. The immunoprecipitation was carried out in 2.0-mm layers of 1% agarose (Litex HAS; Lonza) in HEPES-saline buffer (0.1 M NaCl, 0.05 M HEPES [pH 7.5]) cast on transparent polyester films (Gelbond; Lonza). In most experiments, 15 µl undiluted antiserum was applied to a center well (4 mm), and 15-µl extract samples were applied to the six surrounding wells (4 mm) placed at a distance, edge to edge, of 5 mm. For details, see Fig. 1 and 2. Plates were kept for 2 days at 5°C and then drained, washed, and stained for 10 min (0.5% Coomassie brilliant blue R-250 in ethanol-water-glacial acetic acid [45/45/10 vol/vol/vol]) and rinsed several times afterward in the same solvent until the background was clear. Finally the rinsed plates were dried, and photos were taken.

## SUPPLEMENTAL MATERIAL

Figure S1  Diagrammatic representation of truncated *cps* loci in strains of *S. mitis*, *S. oralis*, and *S. pseudopneumoniae*. Download Figure S1, PDF file, 0.1 MB

Figure S2  Comparison of classical and non-classical *cps2* loci in *S. mitis* SK137 and SK321. Download Figure S2, PDF file, 0.1 MB

Figure S3  Phylogenetic analysis of selected *cps* locus genes. (a) Cluster analysis of nucleotide sequences of *aliB*-like genes in the *cps* locus of streptococci. The *aliC* and *aliD* references are indicated in bold. Note that the *aliD* genes in *S. pneumoniae* serotypes 25F, 25A, and 38 are pseudogenes with numerous premature stop codons and gaps. The number following the strain name is the gene number in the *cps* locus. (b) Phylogenetic analysis of nucleotide sequences of *wzy* genes of *S. pneumoniae* serotypes and commensal streptococci. The linkage specificities of the respective polymerases are as summarized by Bentley et al. (10). Nonpneumococcus strains are indicated by colors (*S. mitis*, green; *S. oralis* subsp. *oralis*, red; *S. oralis* subsp. *tigurinus*, blue; *S. oralis* subsp. *dentisani*, gray; *Streptococcus* sp., cyan). Download Figure S3, PDF file, 0.2 MB

Figure S4  Chemical structure of the SK137 teichoic acid-like capsular polysaccharide and suggested functions of proteins encoded by genes located in its *cps* locus. (A) Structure as reported in reference 16. (B) The same structure presented with a different repeat unit. The Roman numerals I to VII refer to the individual residues. Residues I and VII are the two galactofuranosyl moieties generated by the UDP-galactopyranose mutase encoded by the gene *glf* (SK137_0356). The labels 1 to 2f shown above the structure refer to gene products proposed to be involved in the sequential biosynthetic steps (see Table S2). Download Figure S4, PDF file, 0.2 MB

Figure S5  Diagrammatic representation of the type I restriction modification system (SpnD39III) locus involved in regulation of virulence traits in strains of *S. pneumoniae*, including production of capsular polysaccharide. The comparison with strains of *S. mitis* and *S. oralis* demonstrates its absence in representatives of commensal species. Download Figure S5, PDF file, 0.2 MB

Table S1  Summary of strains and sequences used in the analysis of *cps* loci in commensal streptococci and their characteristics. Table S1, PDF file, 0.4 MB

Table S2  Results of serological analysis by double immunodiffusion of capsular polysaccharides from commensal *Streptococcus* strains with antisera against 12 selected strains and pneumococcal serotypes. Table S2, PDF file, 0.3 MB

Table S3  Annotation of genes in the SK137 cps locus located in the genome between the genes *dexB* and *aliA* Table S3, PDF file, 0.1 MB

Table S4  Capsular biosynthesis loci (*cps*) in selected commensal species of the genus *Streptococcus* Table S4, PDF file, 0.2 MB

Table S5  Proteins encoded by *cps* loci in commensal *Streptococcus* species with no or limited homology to proteins in *S. pneumoniae* Table S5, PDF file, 0.2 MB
