# Supplementary figures and images for: Capsular Polysaccharide Expression in Commensal Streptococcus Species: Genetic and Antigenic Similarities to Streptococcus pneumoniae
Source: mBio. 2016 Nov 15;7(6):e01844-16. doi: 10.1128/mBio.01844-16 (PMC5111408; doi:10.1128/mBio.01844-16)

Fig. S1

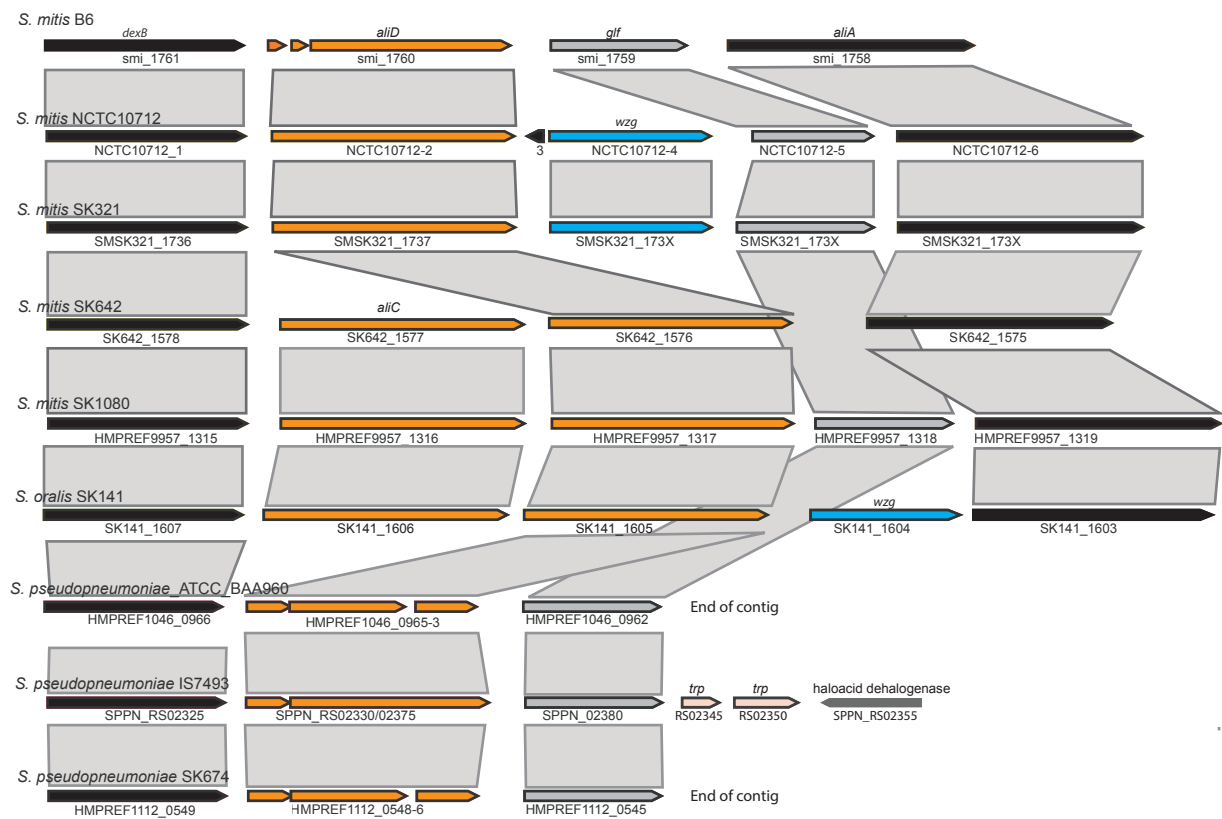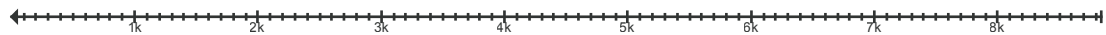

Gene key for *cps* operon genes:

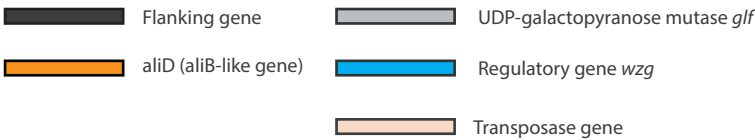

Supplement: Figure S1 — Diagrammatic representation of truncated cps loci in strains of S. mitis, S. oralis, and S. pseudopneumoniae. Download [file mbo006163067sf1.pdf]

Fig. S2

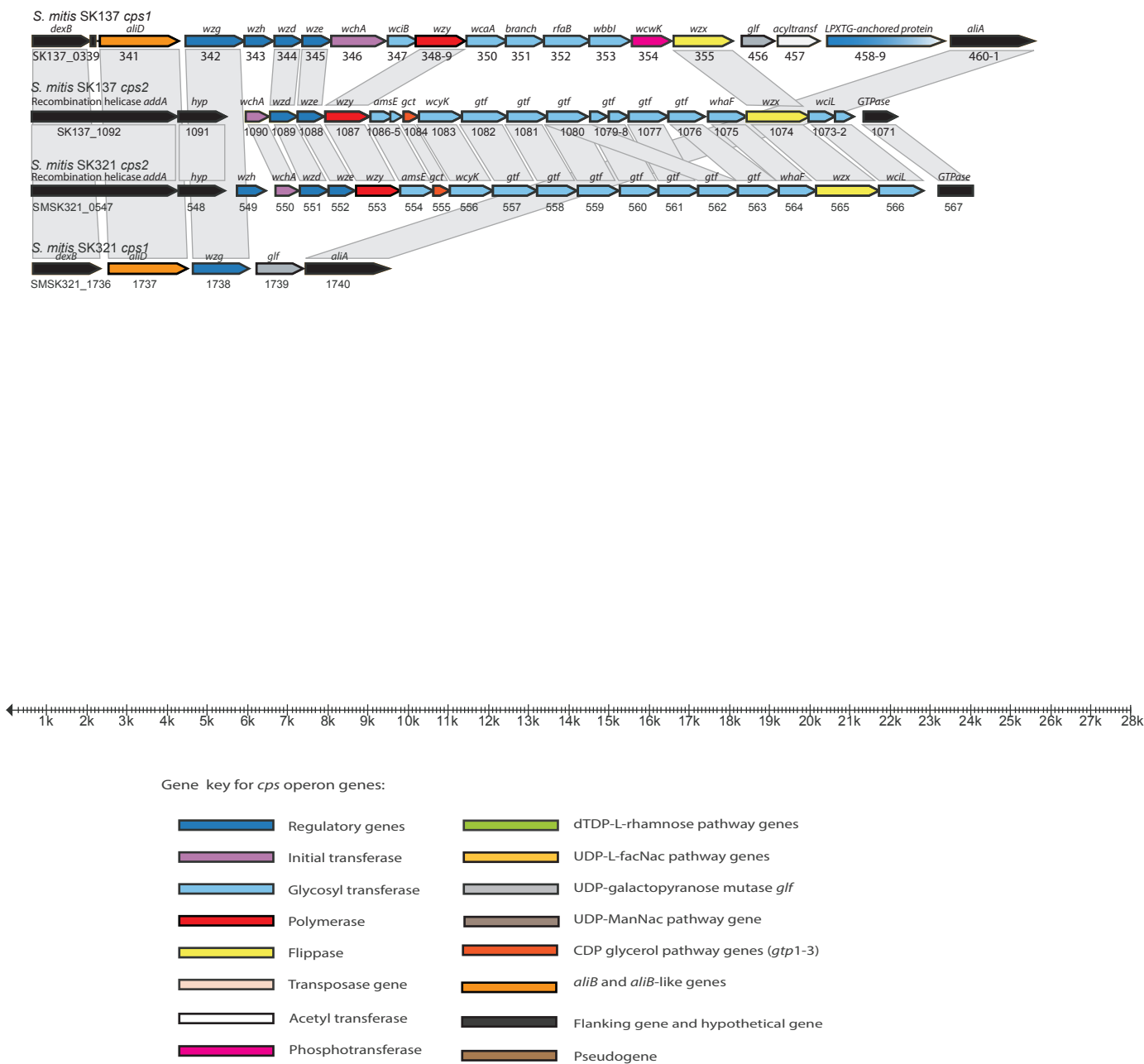

Supplement: Figure S2 — Comparison of classical and non-classical cps2 loci in S. mitis SK137 and SK321. Download [file mbo006163067sf2.pdf]

Fig. S3a

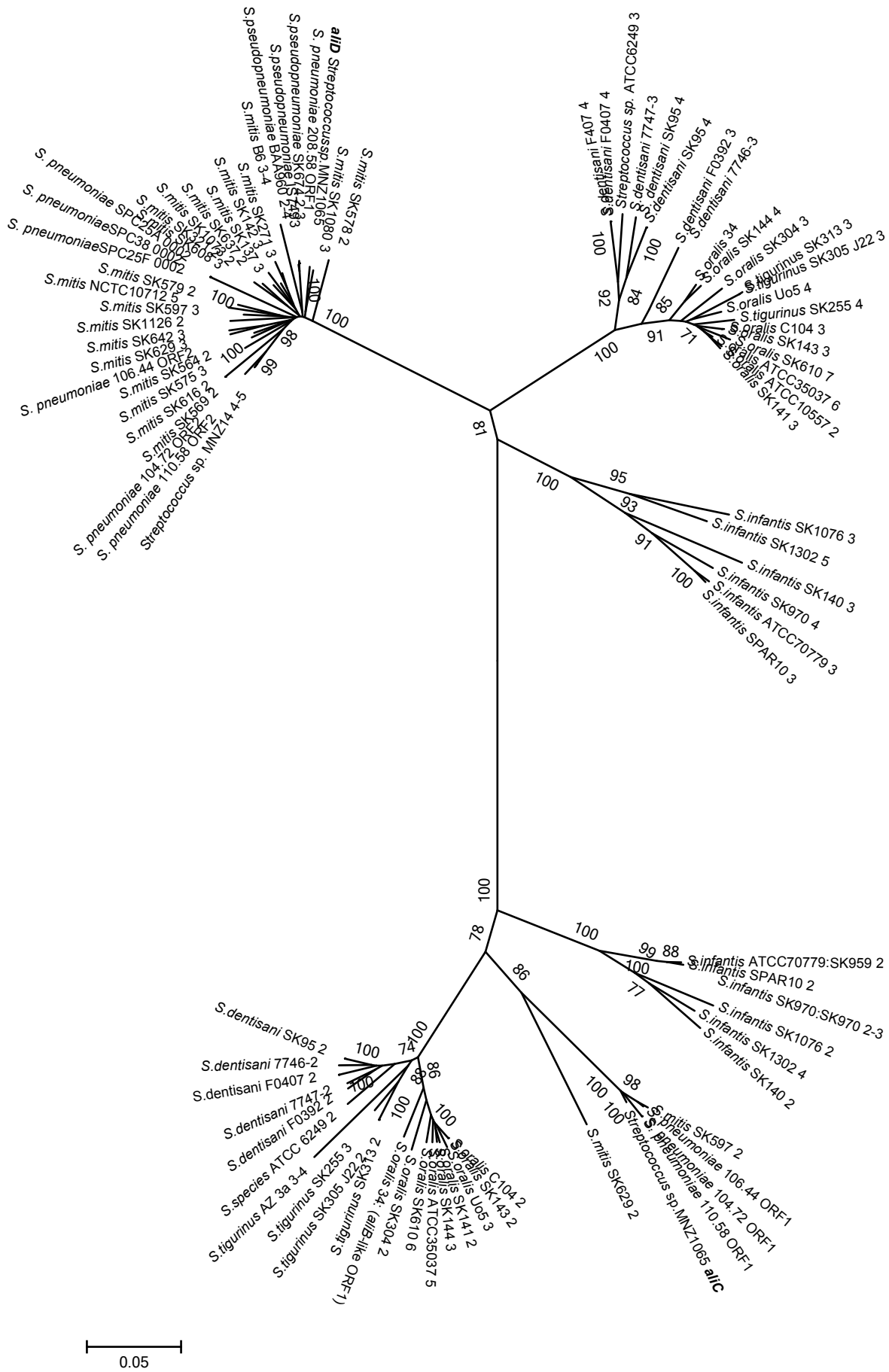

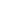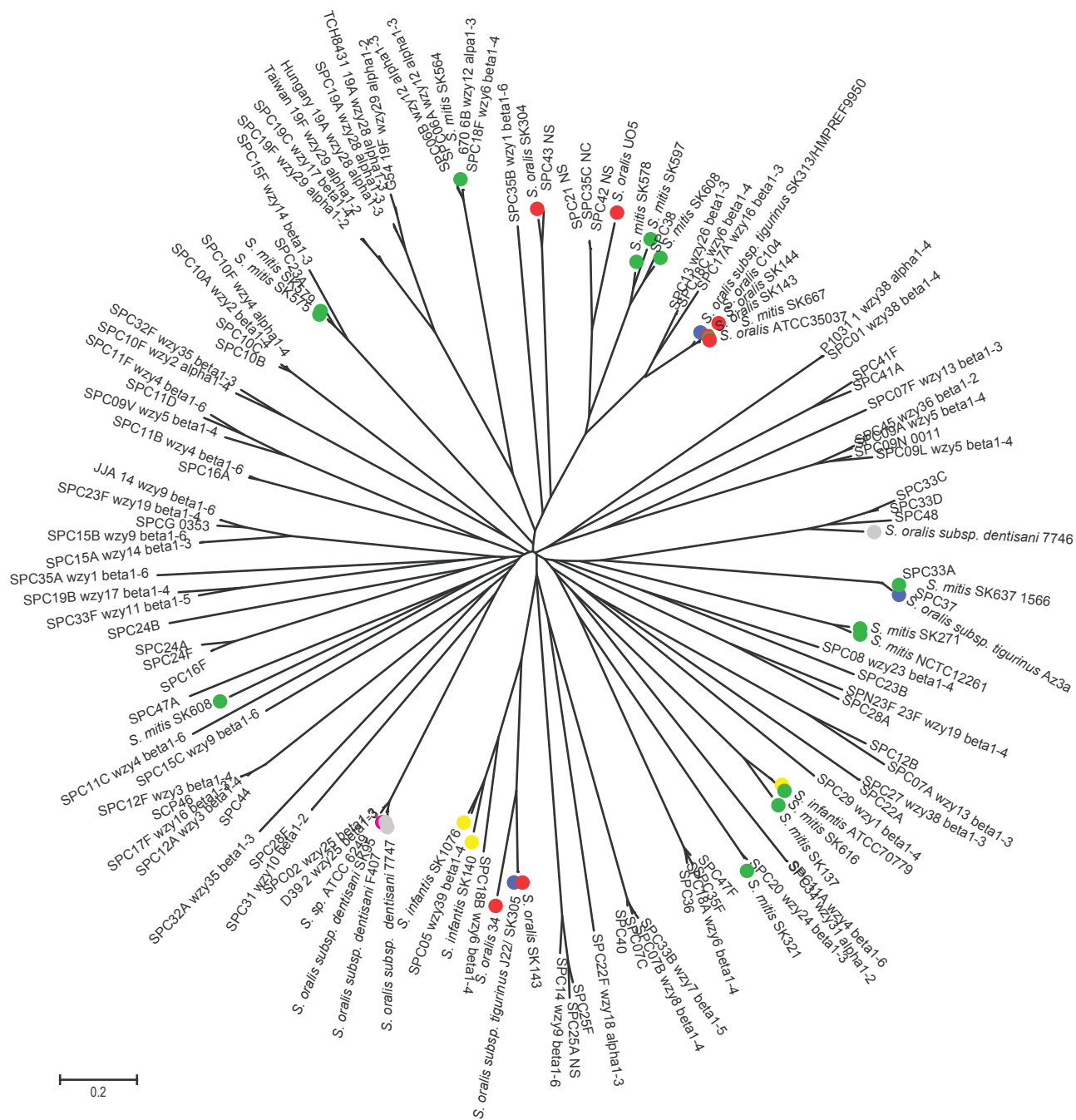

Supplement: Figure S3 — Phylogenetic analysis of selected cps locus genes. (a) Cluster analysis of nucleotide sequences of aliB-like genes in the cps locus of streptococci. The aliC and aliD references are indicated in bold. Note that the aliD genes in S. pneumoniae serotypes 25F, 25A, and 38 are pseudogenes with numerous premature stop codons and gaps. The number following the strain name is the gene number in the cps locus. (b) Phylogenetic analysis of nucleotide sequences of wzy genes of S. pneumoniae serotypes and commensal streptococci. The linkage specificities of the respective polymerases are as summarized by Bentley et al. (10). Nonpneumococcus strains are indicated by colors (S. mitis, green; S. oralis subsp. oralis, red; S. oralis subsp. tigurinus, blue; S. oralis subsp. dentisani, gray; Streptococcus sp., cyan). Download [file mbo006163067sf3.pdf]
