# Supplementary material for: Capsular Polysaccharide Expression in Commensal Streptococcus Species: Genetic and Antigenic Similarities to Streptococcus pneumoniae
Source: mBio. 2016 Nov 15;7(6):e01844-16. doi: 10.1128/mBio.01844-16 (PMC5111408; doi:10.1128/mBio.01844-16)
Supplement: Figure S4 — Chemical structure of the SK137 teichoic acid-like capsular polysaccharide and suggested functions of proteins encoded by genes located in its cps locus. (A) Structure as reported in reference 16. (B) The same structure presented with a different repeat unit. The Roman numerals I to VII refer to the individual residues. Residues I and VII are the two galactofuranosyl moieties generated by the UDP-galactopyranose mutase encoded by the gene glf (SK137_0356). The labels 1 to 2f shown above the structure refer to gene products proposed to be involved in the sequential biosynthetic steps (see Table S2). Download [file mbo006163067sf4.pdf]

A) 6)-β-D-Gal*p*-(1→6)-β-D-Gal*p*-(1→6)-β-D-Gal*f*-(1→3)-β-D-Glc*p*-(1→6)-β-D-Gal*f*-(1→6)-α-D-Gal*p*-(1→6)-α-D-Glc*p*-1-*P*-(O→

| | 2f | 2e | 2d | 2c | 2b | 2a | 1 |
|---|---|---|---|---|---|---|---|
| B) (1→6)-β-D-Gal*f*-(1→6)-α-D-Gal*p*-(1→6)-α-D-Glc*p*-1-*P*-(O→6)-β-D-Gal*p*-(1→6)-β-D-Gal*p*-(1→6)-β-D-Gal*f*-(1→3)-β-D-Glc*p*- | | | | | | | |
| | VII | VI | V | IV | III | II | I |

Figure S4. Chemical structure of the SK137 teichoic acid-like capsular polysaccharide and suggested functions of proteins encoded by genes located in its *cps* locus. A) Structure as reported in reference 16. B) The same structure presented with a different repeat unit. The Roman numbers I-VII refer to the individual residues. Residues I and VII are the two galactofuranosyl moieties generated by the UDP-galactopyranose mutase encoded by the gene *glf* (SK137_0356). The figures 1-2f shown above the structure refer to gene products proposed to be involved in the sequential biosynthetic steps (see Table S2).
