# Supplementary material for: Capsular Polysaccharide Expression in Commensal Streptococcus Species: Genetic and Antigenic Similarities to Streptococcus pneumoniae
Source: mBio. 2016 Nov 15;7(6):e01844-16. doi: 10.1128/mBio.01844-16 (PMC5111408; doi:10.1128/mBio.01844-16)
Supplement: Table S1 — Summary of strains and sequences used in the analysis of cps loci in commensal streptococci and their characteristics. [file mbo006163067st1.pdf]

Table S1. Summary of strains and sequences used in the analysis of *cps* loci in commensal streptococci and their characteristics.

**Species/ Strain**1	**Accession no.**	***cps*** **size**	***S. mitis*** **serology group**	**Homology to** ***S. pneumoniae*** **serotype/group**	**CRP type**	**PCR wzg1-3**	**PCR wzg2-4**	***dexB*** **up-stream**	***aliA*** **down-stream**	***aliB*** **-like**
S. mitis										
**SK137** / CCUG 35791	JPFS01000000	21225	smI			Pos	Pos	Yes	Yes	aliD
		14156 (cps2)		Expression uncertain				No	No	None
**SK597** / CCUG 55094	AEDV00000000	23641	smI			Pos	Pos	Yes	Yes	*aliC + aliD*
**SK608**	PRJNA242555	21468	smI			Pos	Pos	Yes	Yes	*aliD*
**SK142**/NCTC12261T	AEDX00000000	21782	smII			Pos	Pos	Yes	Yes	*aliD*
**SK271**	JPGW00000000	22055	smIII			Pos	Pos	Yes	Yes	*aliD*
SK1073	AFQT00000000	22038	smIII			Pos	Pos	Yes	Yes	*aliD*
**SK564**	AEDU00000000	24612		Serotype 19C		Pos	Pos	Yes	Yes	*aliD*
**SK569** / CCUG 62643	AFUF00000000	23106				Pos	Pos	Yes	Yes	*aliD*
**SK575** / CCUG 62644	AICU00000000	25501		Serotype 45		Pos	Pos	Yes	Yes	*aliD*
SK579	AJJL00000000	25520		Serotype 45		Pos	Pos	Yes	Yes	*aliD*
SK616	AICR00000000	26507		Serotype 45		Pos	Pos	Yes	Yes	*aliD*
**SK611**	No sequence			Serotype 40		Pos	Pos			
**SK637** / CCUG 35816	JPFX00000000	17060	smIV			Pos	Pos	Yes	Yes	*aliD*
SK578	JPFY00000000	16938		Serotype 36		weak	Pos	Yes	Yes	*aliD*
SK1126	JPFT00000000	17161		Serotype 36		Neg	Pos	Yes	Yes	*aliD*
SK667	JPFV00000000	25972		Serogroup 18		Pos	Pos	Yes	yes	*aliD*
SK321	AEDT00000000	5055		No capsule				Yes	Yes	*aliD*
		17185 (cps2)		Expression uncertain		Pos	Pos	No	No	None
NCTC10712/ SK113	PRJNA308845	5293		No capsule		Pos	Pos	Yes	Yes	*aliD*
SK1080	AFQV00000000	5710		No capsule		Neg	Neg	Yes	Yes	*aliC + aliD*
SK629	PRJNA242570	20494		Serotype 33D		Neg	Neg	Yes	Yes	*aliC + aliD*
SK642	PRJNA242572	5408		No capsule		NT	NT	Yes	yes	*aliC + aliD*
B6	FN568063	7513		No capsule		Neg	Neg	Yes	Yes	*aliD*
S. oralis subsp. *oralis*										
NCTC 7864/ ATCC10557/ SK10	AJKO00000000	22303			3G	NT	NT	No	Yes	*aliC + aliD*
**SK23**/ATCC 35037 T	ADMV00000000	24479			3G	NT	NT	No	Yes	*aliC + aliD*
SK141	JPGA00000000	5994				NT	NT	Yes	Yes	*aliC + aliD*
SK143	JPGB00000000	19016			4Gn	NT	NT	Yes	No	*aliC + aliD*
SK144	EF587720	17845			5Gn	NT	NT	No	Yes	*aliC + aliD*
SK304	ALJN00000000	23713		Serotype 16A		NT	NT	Yes	Yes	*aliC + aliD*
SK610	AJKQ00000000	20415			4Gn	NT	NT	Yes	No	*aliC + aliD*
C104	EF587719.1	19021			4Gn	NT	NT	Yes	Yes	*aliC + aliD*
34	AB181234	22277			1Gn	NT	NT	Yes	Yes	*aliC + aliD*
Uo5	NC_015291.1	19291			4Gn	NT	NT	Yes	No	*aliC + aliD*
S. oralis subsp. *dentisani*										
7747 T	CAUK00000000	22180		Serotype 2		NT	NT	No	Yes	*aliC + aliD*
SK95	AFUB00000000	24865		Serotype 2		NT	NT	Yes	Yes	*aliC + aliD*
F0407	AGAE00000000	25018		Serotype 2		NT	NT	Yes	Yes	*aliC + aliD*
F0392	AFUO00000000	21398		Serotype 5		NT	NT	Yes	Yes	*aliC + aliD*
7746	CAUJ00000000	19743				NT	NT	Yes	Yes	*aliC + aliD*
S. oralis subsp. *tigurinus*										
AZ_3a T	AORU01000006	18329		Serotype 33A		NT	NT	Yes	Yes	fragments
HMPREF9950/ SK313/ ATCC15914	AFUU01000001	23269			2G	NT	NT	Yes	Yes	*aliC + aliD*
J22 /SK305	AB181235	23344			2G	NT	NT	Yes	Yes	*aliC + aliD*
SK255 /CCUG 27560	AFNM00000000	18338				NT	NT	Yes	Yes	*aliC + aliD*
SK1074 / CCUG 45494B	AICT01000005	23644		partial 19A		NT	NT	Yes	Yes	*aliC + aliD*
Streptococcus sp.										
ATCC 6249	AEEN00000000	20283				NT	NT	Yes	Yes	*aliC + aliD*
S. infantis										
ATCC 700779 T	AJTA00000000	19031		serotype 36		NT	NT	Yes	No	*aliC + aliD*
SK140	AJML00000000	23289		Serotype 36		NT	NT	Yes	No	*aliC + aliD*
SK1302	AEDY00000000	20353		Serotype 15F rel.		NT	NT	Yes	Yes	*aliC + aliD*
SK1076	AFNN00000000	22149		Serotype 36		NT	NT	Yes	No	*aliC + aliD*
SPAR10	ALCH01000000	21564*		Serotype 10B?		NT	NT	Yes	No	*aliC + aliD*
SK970 /CCUG 36756	AFUT00000000	18075		Serotype 36		NT	NT	Yes	No	*aliC + aliD*
S. pseudopneumoniae										
ATCC BAA-960 T	AICS01000245	3594		No capsule		NT	NT	Yes	?	*aliD* pseudo
IS7493	CP002925	4843		No capsule		NT	NT	Yes	No	*aliD* pseudo
SK674	AJKE00000000	3627		No capsule		NT	NT	Yes	?	*aliD* pseudo

*The *cps* locus sequence includes gap
