# Supplementary material for: Capsular Polysaccharide Expression in Commensal Streptococcus Species: Genetic and Antigenic Similarities to Streptococcus pneumoniae
Source: mBio. 2016 Nov 15;7(6):e01844-16. doi: 10.1128/mBio.01844-16 (PMC5111408; doi:10.1128/mBio.01844-16)
Supplement: Table S2 — Results of serological analysis by double immunodiffusion of capsular polysaccharides from commensal Streptococcus strains with antisera against 12 selected strains and pneumococcal serotypes. [file mbo006163067st2.pdf]

Table S2. Results of serological analysis by double immunodiffusion of capsular polysaccharides from commensal *Streptococcus* strains with antisera against 12 selected strains and pneumococcal serotypes.[1]

		Sero-	Antisera											
Strain	Group	type	SK137	SK142	SK271	SK564	SK569	SK575	SK597	SK608	SK611	SK637	SK1124	SK23[2]
S. mitis														
SK135	smI		+	-	-	-	-	-	+	+	-	-	-	-
SK137	smI		+	-	-	-	-	-	+	+	-	-	-	-
SK138	smI		+	-	-	-	-	-	+	+	-	-	-	-
SK597	smI		+	-	-	-	-	-	+	+	-	-	-	-
SK602	smI		+	-	-	-	-	-	+	+	-	-	-	-
SK608	smI		+	-	-	-	-	-	+	+	-	-	-	-
SK677	smI		+	-	-	-	-	-	+	+	-	-	-	-
SK142	smII		-	+	-	-	-	-	-	-	-	-	-	-
SK271	smIII		-	-	+	-	-	-	-	-	-	-	-	-
SK334	smIII		-	-	+	-	-	-	-	-	-	-	-	-
SK596	smIII		-	-	+	-	-	-	-	-	-	-	-	-
SK1073	smIII		-	-	+	-	-	-	-	-	-	-	-	-
SK564		19C	-	-	-	+	-	-	-	-	-	-	-	-
SK569		19C	-	-	-	-	+	-	-	-	-	-	-	-
SK575		45	-	-	-	-	-	+	-	-	-	-	-	-
SK607		45	-	-	-	-	-	+	-	-	-	-	-	-
SK609		45	-	-	-	-	-	+	-	-	-	-	-	-
SK614		45	-	-	-	-	-	+	-	-	-	-	-	-
SK615		45	-	-	-	-	-	+	-	-	-	-	-	-
SK616		45	-	-	-	-	-	+	-	-	-	-	-	-
SK651		45	-	-	-	-	-	+	-	-	-	-	-	-
SK1122		45	-	-	-	-	-	+	-	-	-	-	-	-
SK611		40	-	-	-	-	-	-	-	-	+	-	-	-
SK637	smIV		-	-	-	-	-	-	-	-	-	+	-	-
SK1123		21	-	-	-	-	-	-	-	-	-	-	+	-
SK1124		21	-	-	-	-	-	-	-	-	-	-	+	-
SK578			-	-	-	-	-	-	-	-	-	-	-	+
SK646			-	-	-	-	-	-	-	-	-	-	-	+
S. oralis														
SK10			-	-	-	-	-	-	-	-	-	-	-	+
SK304		16A	-	-	-	-	-	-	-	-	-	-	-	-
ATCC35037			-	-	-	-	-	-	-	-	-	-	-	+
S. oralis subsp. *dentisani*														
SK95		2	-	-	-	-	-	-	-	-	-	-	-	-
S. infantis														
SK140		36	-	-	-	-	-	-	-	-	-	-	-	-
SK1076		36	-	-	-	-	-	-	-	-	-	-	-	-
S. oralis subsp. *tigurinus*														
SK313			-	-	-	-	-	-	-	-	-	-	-	+

[1]The following strains did not react in any of the antisera: *S. mitis* **B6**, SK24, **SK113/NCTC10712**, SK145, **SK321**, SK322, SK568, SK572, SK574, SK579, SK598, *SK599*, **SK601**, **SK612**, **SK626**, **SK627**, SK629, **SK630**, SK631, SK632, SK634, **SK635**, **SK636**, **SK639**, **SK640**, SK641, **SK642**, **SK648**, **SK649**, **SK650**, **SK653**, SK659, SK661, SK667,SK723, SK1080, SK1125, SK1126, *S. oralis* SK100, SK143, *S. tigurinus* SK255, *S. infantis* ATCC 700779, SK970/CCUG36756 (strains without detectable complete *cps* are indicated in bold).

[2]The reactions in antiserum to SK23 are due to an unidentified antigen distinct from the capsule (see main taxt).
