# Supplementary material for: Capsular Polysaccharide Expression in Commensal Streptococcus Species: Genetic and Antigenic Similarities to Streptococcus pneumoniae
Source: mBio. 2016 Nov 15;7(6):e01844-16. doi: 10.1128/mBio.01844-16 (PMC5111408; doi:10.1128/mBio.01844-16)
Supplement: Table S3 — Annotation of genes in the SK137 cps locus located in the genome between the genes dexB and aliA [file mbo006163067st3.pdf]

Table S3. Annotation of genes in the SK137 *cps* locus located in the genome between the genes *dexB* and *aliA*.

SK137_	Highest scoring homolog [a)]	Step	Suggested function		Description	Occurrence in mitis group streptococci
0340	*dexB*	0	Metabolism	Releases glucose from isomaltosaccharides	Glucan 1,6-α-glucosidase	Highly conserved
0341	*aliD*	7	Regulation	Substrate recognition	"AliB-like", periplasmic peptide-binding protein	Conserved
0342	*Wzg, CpsA;*		Regulation		LytR family transcriptional regulator (super-family)	Conserved
0343	*Wzh, CpsB;*		Regulation		Tyrosine protein phosphatase	Conserved
0344	*Wzd, CpsC;*	6	Cell wall Linkage		Cap. polysaccharide biosynthesis protein (Chain length determinant protein, Wzz??)	Conserved
0345	*Wze, CpsD;*				Tyrosin-protein kinase	Conserved
0346	*WchA, CpsE;*	1	Initiation	UDP-Glc*p*? → UndP	Initial sugar transferase, Exopolysaccharide biosynthesis polyprenyl glycosylphosphotransferase (undecaprenyl phosphate carrier)	Conserved
0347	*WciB, CpsF; - 84%*	2a	Seq. addition step [b)]	D-Gal*f*-1-3-βGlc*p*	Glycosyl transferase (cap33fF, Sv29)	Common bacterial domain in
0348 + 0349	*Wzy, CpsX;*	5	Polymerization	Repeat unit polymerase	Oligosaccharide repeat unit polymerase	No domain recognized, common, less conserved
0350	*WcrM - 47%*	2b	Seq. addition step	D-gal*p*?-1-6-β-D-Gal*f*	Glycosyl transferase [c)] family 2 protein	Common, less conserved
0351	*WcrG - 43%*	2c	Seq. addition step	D-Gal*p*-1-6-β-D-Gal*p*?	Core-2/I-branching enzyme? β-1,6-N-acetylglucosaminyltransferase?	Common, less conserved
0352	*WciL- 35%, WbnE?;*	2e?	Seq. addition step	Uncertain	Glycosyl transferase family 4 = SK137_0352? GT1_CapH_like, a predicted retaining α-glycosyl transferases	Diverse,
0353	*WcrH - 38%*	2f	Seq. addition step	D-Gal*f*-1-6-α-D-Gal*p*	Galactofuranosyl transferase	Common, less conserved
0354	*WcwK - 45% CapG*	2d	Seq. addition step	α-D-Glc*p*-1-P-6-β-D-Gal*p*	Capsular polysaccharide phosphotransferase, α-Glc*p*-1P transferase	Common, less conserved. No domain recognized.
0355	*Wzx*	3	Transfer	Repeat unit	Flippase	Conserved
0356	*Glf*	4	Galactofuranose biosynthesis	UDP-Gal*p* → UDP-Gal*f*	UDP-galactopyranose mutase	Common, conserved
0357	*WcjE – 45%*	2g?			Acetyltransferase – nonfunctional???	Common, less conserved
0358	*TolA*	?	Unknown	-	Peptidase? secreted protein?	Not common, not conserved
0359	*None detected*	?	Unknown	Protease?	Contain a LPXTG- C-terminal cell wall anchoring motif and a G5 domain. Possible a cell wall surface protein with protease activity.	Not common, less conserved
0360	*AliA*	0			oligopeptide-binding protein	

a) Designation in *S. pneumoniae*?? (% amino acid identity/similarity)
b) Seq. = sequential
c) Glycosyltransferases catalyze the transfer of sugar moieties from activated donor molecules to specific acceptor molecules.
