# Supplementary material for: Capsular Polysaccharide Expression in Commensal Streptococcus Species: Genetic and Antigenic Similarities to Streptococcus pneumoniae
Source: mBio. 2016 Nov 15;7(6):e01844-16. doi: 10.1128/mBio.01844-16 (PMC5111408; doi:10.1128/mBio.01844-16)
Supplement: Table S4 — Capsular biosynthesis loci (cps) in selected commensal species of the genus Streptococcus [file mbo006163067st4.pdf]

Table S4. Capsular biosynthesis loci (*cps*) in 14 selected strains of commensal species of the genus *Streptococcus*[1].

Species	Strain	Accession #	Coordinates	Comments
S. anginosus	SK52[T]	AFIM01000039.1	11518-18891 (end of contig)	
S. anginosus	SK1138	ALJO01000004.1	47590-30756	
S. constellatus	SK53[T]	AICQ01000046.1	57895-?	transposases
S. constellatus	KCOM 1650	JWIY01000004.1	147926-174163	transposases
S. intermedius	SK54[T]	BASV01000014.1	8619-1 (beginning of contig)	
S. cristatus	ATCC 51100	AEVC01000024.1	91663-100544 (end of contig)	
S. cristatus	CR3	JYGK01000003.1	145985-166584	
S. cristatus	CC5A	JYGJ01000006.1	161741-179875	
S. parasanguinis	SK236	AFUC01000012.1	70338-86785	
S. parasanguinis	F0449	AJMV01000016.1	17211-1 (beginning of contig)	
S. australis	ATCC 700641	AFUD01000011.1	56219-40884	*wze* missing
S. gordonii	Challis/CH1	CP000725.1	2095117-2076638	transposases
S. gordonii	CCUG 33482	LQWV01000034.1	65457-81837 (end of contig)	
S. gordonii	38	AY147914.1	1088-22907	

[1] In all strains the start of the *cps* locus was flanked by a gene encoding an anaerobic ribonucleoside-triphosphate reductase activating protein.
