# Supplementary material for: Capsular Polysaccharide Expression in Commensal Streptococcus Species: Genetic and Antigenic Similarities to Streptococcus pneumoniae
Source: mBio. 2016 Nov 15;7(6):e01844-16. doi: 10.1128/mBio.01844-16 (PMC5111408; doi:10.1128/mBio.01844-16)
Supplement: Table S5 — Proteins encoded by cps loci in commensal Streptococcus species with no or limited homology to proteins in S. pneumoniae [file mbo006163067st5.pdf]

Table S5. Proteins encoded by *cps* loci in commensal *Streptococcus* species with no or limited homology to proteins in *S. pneumoniae*.[1]

Strain	Locus	Annotation
Streptococcus sp. ATCC 6249	ATCC6249_10	ABC superfamily ATP binding cassette transporter permease protein
S. oralis SK144	SK144_2	Acetyltransferase family protein
S. mitis SK637	SK637_14	Acyltransferase family protein
S. mitis SK629	SK629_17	Acyltransferase family protein
S. oralis C104	C104_1	alpha amylase catalytic domain protein
S. mitis SK608	SK608_16	Capsular polysaccharide phosphotransferase
S. mitis SK597	SK597_15	Capsular polysaccharide phosphotransferase
S. mitis SK137	SK137_16	Capsular polysaccharide phosphotransferase
S. mitis SK597	SK597_12	Core2/IBranching enzyme family protein
S. mitis SK137	SK137_13	Core2/IBranching enzyme family protein
S. mitis SK608	SK608_13	Core2/IBranching enzyme family protein
Streptococcus sp. ATCC 6249	ATCC6249_14	Cytidyltransferase-like domain protein
S. mitis SK667	SK667_25	dTDP-4-dehydrorhamnose reductase
S. mitis SK616	SK616_24	FAD dependent oxidoreductase family protein
S. mitis SK637	SK637_15	Flippase mviNlike family protein
S. oralis subsp. *tigurinus* SK313	SK313_18	Flippase mviNlike family protein
S. mitis SK137	SK137_20-21	LPXTG-motif cell wall anchor domain protein (G5 domain protein)
S. mitis SK597	SK597_13	Glycosyl transferases group 1 family protein
S. mitis SK608	SK608_14	Glycosyl transferases group 1 family protein
S. mitis SK137	SK137_14	Glycosyl transferases group 1 family protein
S. mitis SK137	SK137_15	Glycosyl transferases group 1 family protein
S. mitis SK608	SK608_15	Glycosyl transferases group 1 family protein
S. mitis SK597	SK597_14	Glycosyl transferases group 1 family protein
S. oralis subsp. *tigurinus* SK313	SK313_13	Glycosyl transferases group 1 family protein
S. mitis NCTC12261/SK142	SM12261_0996	Glycosyl transferase group 2 family protein
S. mitis NCTC12261/SK142	SM12261_0997	Glycosyl transferase group 2 family protein
S. mitis SK578	SK578_12	Glycosyl transferase group 2 family protein
S. mitis SK137	SK137_12	Glycosyl transferase group 2 family protein
S. oralis SK10 /ATCC 15557	SK10_11	Glycosyl transferase group 2 family protein
S. oralis SK10 / ATCC 15557	SK10_13	Glycosyl transferase group 2 family protein fragment
S. oralis SK143	SK143_9	Glycosyl transferase
Streptococcus sp. ATCC 6249	ATCC6249_12	licD family protein (transferase)
S. mitis SK142	SM12261_1001	N-acetylglucosamine-1-phosphotransferase subunits alpha/beta
S. infantis SK1073	SK1073_15	N-acetylglucosamine-1-phosphotransferase subunits alpha/beta
S. mitis SK271	SK271_18	N-acetylglucosamine-1-phosphotransferase subunits alpha/beta
S. mitis SK271	SK271_15	O antigen ligase like membrane family protein
S. mitis SK137	SK137_10-11	Oligosaccharide repeat unit polymerase Wzy
S. mitis SK608	SK608_10	Oligosaccharide repeat unit polymerase Wzy

S. mitis SK1126	SK1126_13	Oligosaccharide repeat unit polymerase Wzy
S. oralis SK10 / ATCC 15557	SK10_12	Oligosaccharide repeat unit polymerase wzy
S. oralis SK10 /ATCC 15557	SK10_14	Oligosaccharide repeat unit polymerase wzy
S. infantis ATCC70779	HMPREF9423_1892	Oligosaccharide repeat unit polymerase wzy
S. mitis SK629	SK629_16	Oligosaccharide repeat unit polymerase wzy
S. mitis SK597	SK597_10	Oligosaccharide repeat unit polymerase wzy
S. oralis SK144	SK144_11	Oligosaccharide repeat unit polymerase wzy
S. mitis NCTC12261/SK142	SM12261_998	Oligosaccharide repeat unit polymerase wzy
S. oralis subsp. *tigurinus* SK313	SK313_16	Oligosaccharide repeat unit polymerase wzy

[1] Non-pneumococcus Mitis group *cps* locus proteins that do not have matches above 50% amino acid sequence identity over 30% of the length of *S. pneumoniae* proteins encoded in the *cps* locus.
