# Supplementary material for: Capsular Polysaccharide Expression in Commensal Streptococcus Species: Genetic and Antigenic Similarities to Streptococcus pneumoniae
Source: mBio. 2016 Nov 15;7(6):e01844-16. doi: 10.1128/mBio.01844-16 (PMC5111408; doi:10.1128/mBio.01844-16)
Supplement: Figure S5 — Diagrammatic representation of the type I restriction modification system (SpnD39III) locus involved in regulation of virulence traits in strains of S. pneumoniae, including production of capsular polysaccharide. The comparison with strains of S. mitis and S. oralis demonstrates its absence in representatives of commensal species. Download [file mbo006163067sf5.pdf]

Figure S5

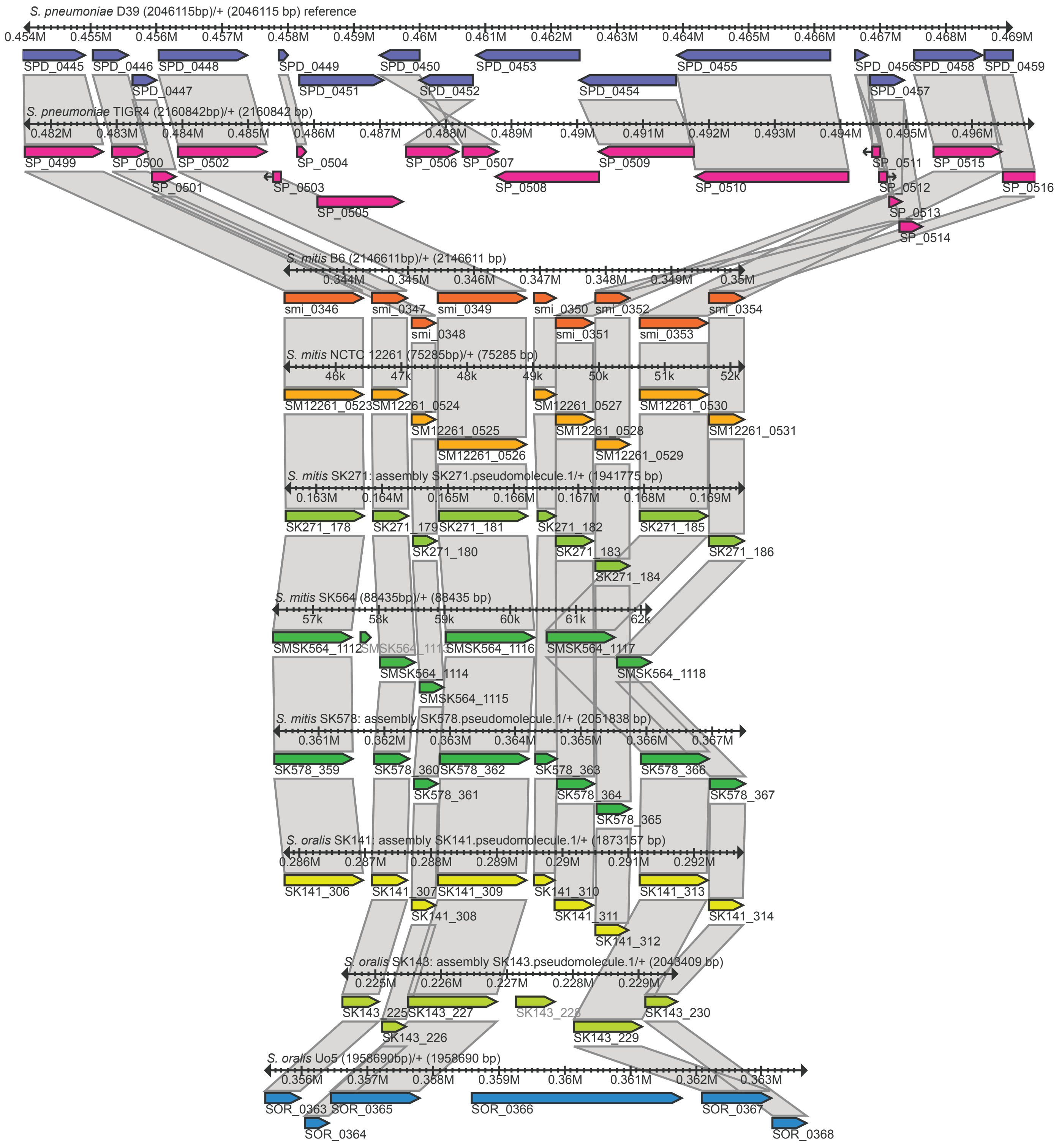

S. pneumoniae D39 (2046115bp)/+ (2046115 bp) reference
0.454M 0.455M 0.456M 0.457M 0.458M 0.459M 0.46M 0.461M 0.462M 0.463M 0.464M 0.465M 0.466M 0.467M 0.468M 0.469M
SPD_0445 SPD_0446 SPD_0447 SPD_0448 SPD_0449 SPD_0450 SPD_0451 SPD_0452 SPD_0453 SPD_0454 SPD_0455 SPD_0456 SPD_0457 SPD_0458 SPD_0459
S. pneumoniae TIGR4 (2160842bp)/+ (2160842 bp)
0.482M 0.483M 0.484M 0.485M 0.486M 0.487M 0.488M 0.489M 0.49M 0.491M 0.492M 0.493M 0.494M 0.495M 0.496M
SP_0499 SP_0500 SP_0501 SP_0502 SP_0503 SP_0504 SP_0505 SP_0506 SP_0507 SP_0508 SP_0509 SP_0510 SP_0511 SP_0512 SP_0513 SP_0514 SP_0515 SP_0516
S. mitis B6 (2146611bp)/+ (2146611 bp)
0.344M 0.345M 0.346M 0.347M 0.348M 0.349M 0.35M
smi_0346 smi_0347 smi_0348 smi_0349 smi_0350 smi_0351 smi_0352 smi_0353 smi_0354
S. mitis NCTC 12261 (75285bp)/+ (75285 bp)
46k 47k 48k 49k 50k 51k 52k
SM12261_0523 SM12261_0524 SM12261_0525 SM12261_0526 SM12261_0527 SM12261_0528 SM12261_0529 SM12261_0530 SM12261_0531
S. mitis SK271: assembly SK271.pseudomolecule.1/+ (1941775 bp)
0.163M 0.164M 0.165M 0.166M 0.167M 0.168M 0.169M
SK271_178 SK271_179 SK271_180 SK271_181 SK271_182 SK271_183 SK271_184 SK271_185 SK271_186
S. mitis SK564 (88435bp)/+ (88435 bp)
57k 58k 59k 60k 61k 62k
SMSK564_1112 SMSK564_1113 SMSK564_1114 SMSK564_1115 SMSK564_1116 SMSK564_1117 SMSK564_1118
S. mitis SK578: assembly SK578.pseudomolecule.1/+ (2051838 bp)
0.361M 0.362M 0.363M 0.364M 0.365M 0.366M 0.367M
SK578_359 SK578_360 SK578_361 SK578_362 SK578_363 SK578_364 SK578_365 SK578_366 SK578_367
S. oralis SK141: assembly SK141.pseudomolecule.1/+ (1873157 bp)
0.286M 0.287M 0.288M 0.289M 0.29M 0.291M 0.292M
SK141_306 SK141_307 SK141_308 SK141_309 SK141_310 SK141_311 SK141_312 SK141_313 SK141_314
S. oralis SK143: assembly SK143.pseudomolecule.1/+ (2043409 bp)
0.225M 0.226M 0.227M 0.228M 0.229M
SK143_225 SK143_226 SK143_227 SK143_228 SK143_229 SK143_230
S. oralis Uo5 (1958690bp)/+ (1958690 bp)
0.356M 0.357M 0.358M 0.359M 0.36M 0.361M 0.362M 0.363M
SOR_0363 SOR_0364 SOR_0365 SOR_0366 SOR_0367 SOR_0368
